# City composition and accessibility statistics in and around Paris

**DOI:** 10.3389/fdata.2024.1354007

**Published:** 2024-03-01

**Authors:** Marie-Olive Thaury, Simon Genet, Léopold Maurice, Paola Tubaro, Sarah J. Berkemer

**Affiliations:** ^1^ENSAE, Institut Polytechnique de Paris, Palaiseau, France; ^2^CREST CNRS-ENSAE, Institut Polytechnique de Paris, Palaiseau, France; ^3^LIX CNRS UMR 7161, École Polytechnique, Institut Polytechnique de Paris, Palaiseau, France; ^4^Earth-Life Science Institute, Tokyo Institute of Technology, Tokyo, Japan

**Keywords:** X-min city, accessibility statistics, city composition, urban modeling, Paris, *OpenStreetMap*

## Abstract

**Introduction:**

Is Paris a 15-min city, where inhabitants can access essential amenities such as schools and shops with a 15-min walk or bike ride? The concept of a 15-min (more generally, X-minute) city was launched in the French capital and was part of the current mayor's plan in her latest re-election campaign. Yet, its fit with the existing urban structure had not been previously assessed.

**Methods:**

This article combines open map data from a large participatory project and geo-localized socio-economic data from official statistics to fill this gap.

**Results:**

We show that, while the city of Paris is rather homogeneous, it is nonetheless characterized by remarkable inequalities between a highly accessible city center (though with some internal differences in terms of types of amenities) and a less well-equipped periphery, where lower-income neighborhoods are more often found. The heterogeneity increases if we consider Paris together with its immediate surroundings, the "Petite Couronne," where large numbers of daily commuters and other users of city facilities live.

**Discussion:**

We thus conclude that successful implementation of the X-minute-city concept requires addressing existing socio-economic inequalities, and that especially in big cities, it should be extended beyond the narrow boundaries of the municipality itself to encompass the larger area around it.

## 1 Introduction

Is Paris a 15-min city? Launched precisely in the French capital, the 15-min (or more generally, X-min) city concept (Moreno, 2016) reflects the urban planning objective of giving inhabitants access to essential amenities within a X-min walk or bike ride. Living in a 15-min city—a polycentric city—is expected to improve personal health and sociability, ensure sustainability, and support the fight against climate change (Moreno et al., 2021, 2023). The objective endorsed by the Mayor of Paris, Anne Hidalgo, during her 2020 electoral campaign, is to reduce carbon emissions from mobility while promoting physical activity.^1^ Paris was a finalist for the World Resources Institute Ross Center Prize for Cities in 2021–2022.^2^ However, there is very limited scientific evidence on the extent to which Paris fulfills the 15-min city criteria so far.

**Figure 1:**
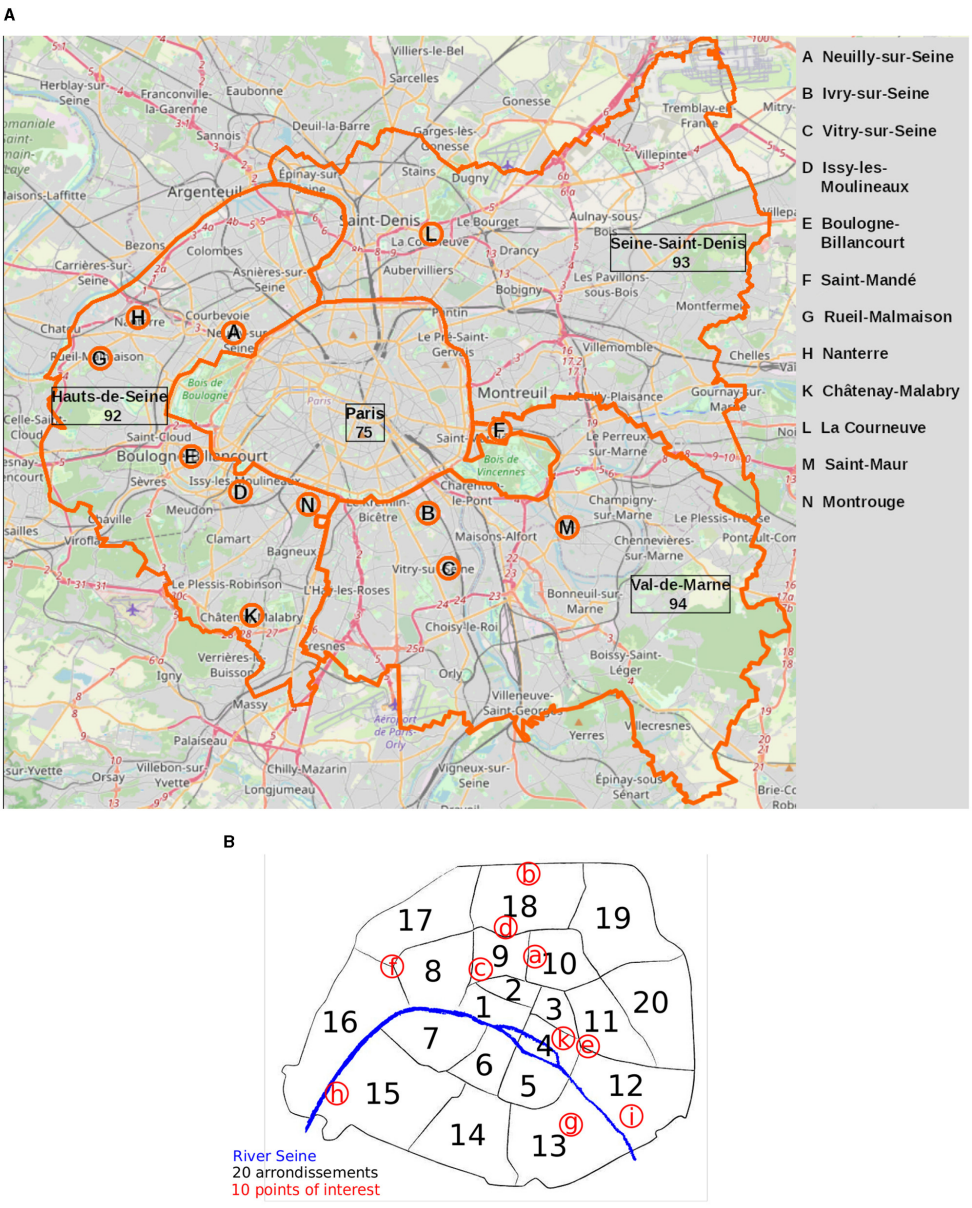
**(A)** The *Petite Couronne* includes Paris (75) and its immediate suburbs, made up of the three departments bordering the capital: Hauts-de-Seine (92), Seine-Saint-Denis (93), and Val-de-Marne (94). It includes more than a hundred towns. The labeled towns will be mentioned throughout our analysis. **(B)** Map of Parisian districts (arrondissements, or short “arr”) officially numbered from 1 to 20 and the POIs mentioned in Figure 5, hence: a Gare du Nord; b Porte de la Chapelle; c Opéra Garnier; d Sacré Coeur; e Place de la Bastille; f Arc de triomphe; g Olympiades; h Quartier de Javel; i Église du Saint-Esprit; k Place des Vosges. Empty map taken from: fr.map-of-paris.com.

At the same time, Paris constitutes a very diverse and unequal space. It is the second most unequal French city in terms of income according to *Observatoire des inégalités*, preceded only by nearby Neuilly-sur-Seine, located just west of world-famous *Champs Élysées*, as depicted Figure 1A. The ratio between the minimum income of the wealthiest 10% and the maximum income of the poorest 10% in Paris is 6.4 compared to 3.4 for France as a whole (excluding overseas regions).^3^ There are also large disparities across neighborhoods in terms of urban functions, with for example large and intermodal hubs at the Halles, shopping streets around the Opéra, and more residential areas in the 16th *arrondissement* (district). Taking into account the whole area around Paris brings to light an even more extreme degree of polarization, with hubs of poverty in the north-east of the city (the *départment* of Seine-Saint-Denis, see Figure 1A) and in specific towns such as Ivry-sur-Seine and Vitry-sur-Seine (**B** and **C** in Figure 1A, respectively); and poles of wealth such as the just mentioned Neuilly-sur-Seine (Pinçon and Pinçon-Charlot, 2015; Clerval, 2016).

This article evaluates Paris in light of the 15-min city concept while taking into account the large socio-economic inequalities among its inhabitants. We build and analyze accessibility measures for inner Paris alone and for Paris with its immediate surroundings, called *Petite Couronne* and depicted in Figure 1A. The *Petite Couronne* includes Paris (75) and its immediate suburbs, made up of three departments bordering the capital: Hauts-de-Seine (numbered 92), Seine-Saint-Denis (93), and Val-de-Marne (94). It includes more than a hundred towns. Figure 1B shows Paris, its districts numbered from 1 to 20 (called *arrondissements*) and some points-of-interest (POI) analyzed in this study.^4^ The shape of Paris differs slightly across figures as the most Eastern and most Western extensions of its city boundary frame its biggest green spaces, the *Bois de Boulogne* (West) and the *Bois de Vincennes* (East). Sparsely endowed with amenities, these areas are often excluded from analyses and maps.

Since its introduction in 2016, the X-min city concept has gained increasing popularity, and a diverse set of methods to measure accessibility of amenities and distances to POIs have been introduced. Here, we summarize insights from the recent studies that are more directly relevant to the work we intend to undertake. Studies of the 15-min city concept for Barcelona (Ferrer-Ortiz et al., 2022), the 20-min city concept for Greater Liverpool (Calafiore et al., 2022), an overview of 15-min city concepts in various European cities (Bartzokas-Tsiompras and Bakogiannis, 2023), and a comparison of US cities (Logan et al., 2022) use accessibility measures based on the distances within the area of study, focusing on walking or cycling. An analysis of the 15-min city concept in Italy included additional city-specific data in order to gain more information about the places (Olivari et al., 2023). As a drawback, the study is not easily extendable to other cities, as each city or country may collect distinct data sets that are not always available elsewhere. A comparison of 15-min city concepts in Paris, Rome, and London (Barbieri et al., 2023) used a graph structure in order to represent the cities and analyze the accessibility of amenities. This leads to very detailed and accurate distance measures regarding the study area, however the analysis is very resource intensive and thus, does not allow to include further data sets and has large computational requirements. In turn, Birkenfeld et al. (2023) claim that 15-min city concepts are not easily applicable everywhere, depending on people's lifestyle as well as the environment and city architecture. In particular, they are infeasible in North America, where according to the authors, a 30-min concept is more likely to be successfully implemented.

To develop our analysis, we build on two recent studies that developed and implemented measures of accessibility to shops and essential local services in order to assess possible inequalities between different areas, particularly between the city and the suburbs. In 2020, INSEE^5^ published a study (Cazaubiel and Cohen, 2020) in which researchers implemented the 2SFCA score (Luo and Wang, 2003), which will be detailed later, to evaluate individuals' access to shops throughout the country. They also used the distance between individuals and shops as well as data from a family budget [*Budget de famille (Bdf)*] survey to measure the preference of households for local shops. This study reveals a strong inequality of access between households living in the centers of large urban areas and those living on the outskirts, since 20% of the latter have poor access to essential retail, compared to 0.4% of the former. However, the model assumes a distance of 20 km, which can be achieved in 1 day by car and not by foot or bicycle. Therefore, it is useful to account for possible inequalities in large regions but not within cities. On top of showing that the countryside has fewer shops per capita than other cities, an earlier INSEE study (Trevien, 2017) did highlight differences within large cities. As a matter of fact, by calculating the distance as the crow flies^6^ between households and shops, they showed that the population density of the neighborhood is a major factor in the proximity of shops and that butchers' shops are more accessible in modest districts, and fishmongers in well-to-do districts. We will seek to verify some of these results in our study using the 2SFCA and spatial regression.

We also draw inspiration from a recent evaluation of the 10 and 15-min city concepts in Utrecht, Netherlands (Knap et al., 2023). The authors build an accessibility score per amenity type based on infrastructure (roads, bike network), residents' behaviors (cycling speed, age), a distance decay function (the further away the amenity is, the less likely it is to be visited), and the demand for this amenity (higher demand meaning lower accessibility). They also create a X-min global accessibility score: they make a service-need weighted sum of the accessibility scores of each type of amenity (e.g., food supply is more important than restaurants, and will therefore weigh more). Accessibility scores used in this article are an application of the 2SFCA method. The authors show that people living in the city center of Utrecht have a higher 10-min score than those living in the peripheries. Their results are based on spatial regression models on socio-economic variables such as the percentage of people receiving the minimum income in the neighborhood. For instance, they found a negative relationship between the 10-min score and the percentage of the population below 15, which means that households with children have poorer access to amenities and shops compared to childless households.

Within this study, we use the 2SFCA score together with socio-economic variables provided by the INSEE *Filosofi* database and mapping data of *OpenStreetMap* (OSM) as described in Section 2.1. Similar to the study of Knap et al. (2023), we use the data to analyze accessibility to essential amenities in Paris and its suburbs in the *Petite Couronne*. Analyzing the area around chosen POIs shows differences in city composition for different districts as described in Section 3.1. We calculate the aggregated 2SFCA score for the complete study area as shown in Section 3.2. Results of our regression analysis described in Section 3.3 and the clustering regarding accessibility score and analysis of confounding factors (Section 3.4) underline our findings and confirm inequalities within Paris and between Paris and its suburbs. Figures 1A, B hereby serve as an orientation to the reader regarding the location of towns and districts mentioned in the analysis.

## 2 Materials and methods

We used data downloaded and extracted in March and April 2023. The data sets have been used as provided. More details on materials and methods are available in the Supplementary material.

### 2.1 Data sets

Our analysis is mainly based on two data sources: mapping data from *OpenStreetMap* and a national French set on economic data called *Filosofi*.

#### 2.1.1 *OpenStreetMap*

We use data from *OpenStreetMap* (OSM, osm.org), a large participatory project to collect and share spatial information such as buildings, transportation networks and POI data. Just like *Wikipedia*, the development of OSM is collaborative such that users can enter and edit information. OSM is based on a network of individual nodes where each node is classified into certain categories regarding infrastructure or landscape such as street, building, or river. Furthermore, POIs are divided into several classes (public, health, leisure, catering, accommodation, shopping, money, tourism, etc.), which are themselves subdivided into tags. For example, OSM distinguishes bars from restaurants and fast-food outlets. OSM is well-documented and further information on tags and underlying data structures can be found in the OSM wiki pages (wiki.openstreetmap.org).

#### 2.1.2 Combination with socio-economic data from *Filosofi*

To carry out our various analyzes, we use data from the *Filosofi*^7^ system (French localized fiscal and social income). This INSEE database divides the territory into squares of 200*m*×200*m*, thus overcoming administrative boundaries. It provides, among others, variables such as the age pyramid of the inhabitants, their income and the year of construction of the buildings.

#### 2.1.3 Choice of categories

In order to analyze the city composition around POIs, we define aggregated categories of services, based on *OpenStreetMap* tags:

- **Restaurants**: all types of restaurants including cafes, bars, fast-foods, pubs, and ice-cream shops.
- **Culture and art**: shops and amenities related to literature, music, cinema, plastic arts, performances, video games, games.
- **Education**: primary schools, middle schools, high schools, colleges, and universities.
- **Food shops**: including supermarkets as well as specialist food shops (e.g., bakeries, butchers, dairy shops, seafood shops, wine shops, etc.).
- **Fashion and beauty:** all shops related to clothes, fashion accessories (e.g., jewelry, watches), beauty care (e.g., cosmetics, hairdresser, massage, hair removal, and perfumery).
- **Supply shops:** everyday life shops apart from food shops (e.g., insurance, sport shops, furniture shops, household appliance shops, etc.).

Details of the *OpenStreetMap* tags in each category are listed in Supplementary Table 1. For further details on OSM tags, we refer the reader to the OSM wiki pages for shops^8^ and amenities.^9^

### 2.2 Methods

To unpack the composition of Paris and its suburbs as well as to confirm our findings, we perform the following steps.

1. We first compute basic descriptive statistics to analyze and compare the composition of Paris at the scale of neighborhoods organized around a central location.
2. In a second step, we use accessibility scores to obtain a more global analysis of the city of Paris.
3. We calculate the aggregated 2SFCA score for our chosen categories for the complete area of the *Petite Couronne*, both with and without inner Paris in order to assess the extent to which it drives the calculation of the score.
4. We build on the results of our regression analysis to confirm the outcome of our accessibility measurements for Paris and its suburbs.
5. As a last step, we perform a clustering analysis, again on Paris and its suburbs, including and excluding the city itself, to display groups of similar neighborhoods.

The core of our analysis, around which all our methods revolve, is the calculation of the accessibility score (aggregated 2SFCA).

#### 2.2.1 2SFCA and accessibility measures

Inspired by Knap et al. (2023) and the policy plans of the Mayor of Paris, we focus on the composition of cities driven by services accessibility in a walking distance of 15 min, which roughly corresponds to a 1-km radius at a 5 km/h speed as defined in the OECD accessibility framework of cities.^10^

Accessibility scores are an application of the *two-step floating catchment area method* (2SFCA; Luo and Wang, 2003). It has been first used to measure health services accessibility, but it can be applied to any sort of extensive variables like the number of amenities for instance. The idea behind 2SFCA is to measure for each service provider the surrounding demand. Then, as a second step, for each person (or place) asking for this service, we calculate the surrounding supply by considering that each service provider divides itself up on the previously calculated demand.

**Figure 2:**
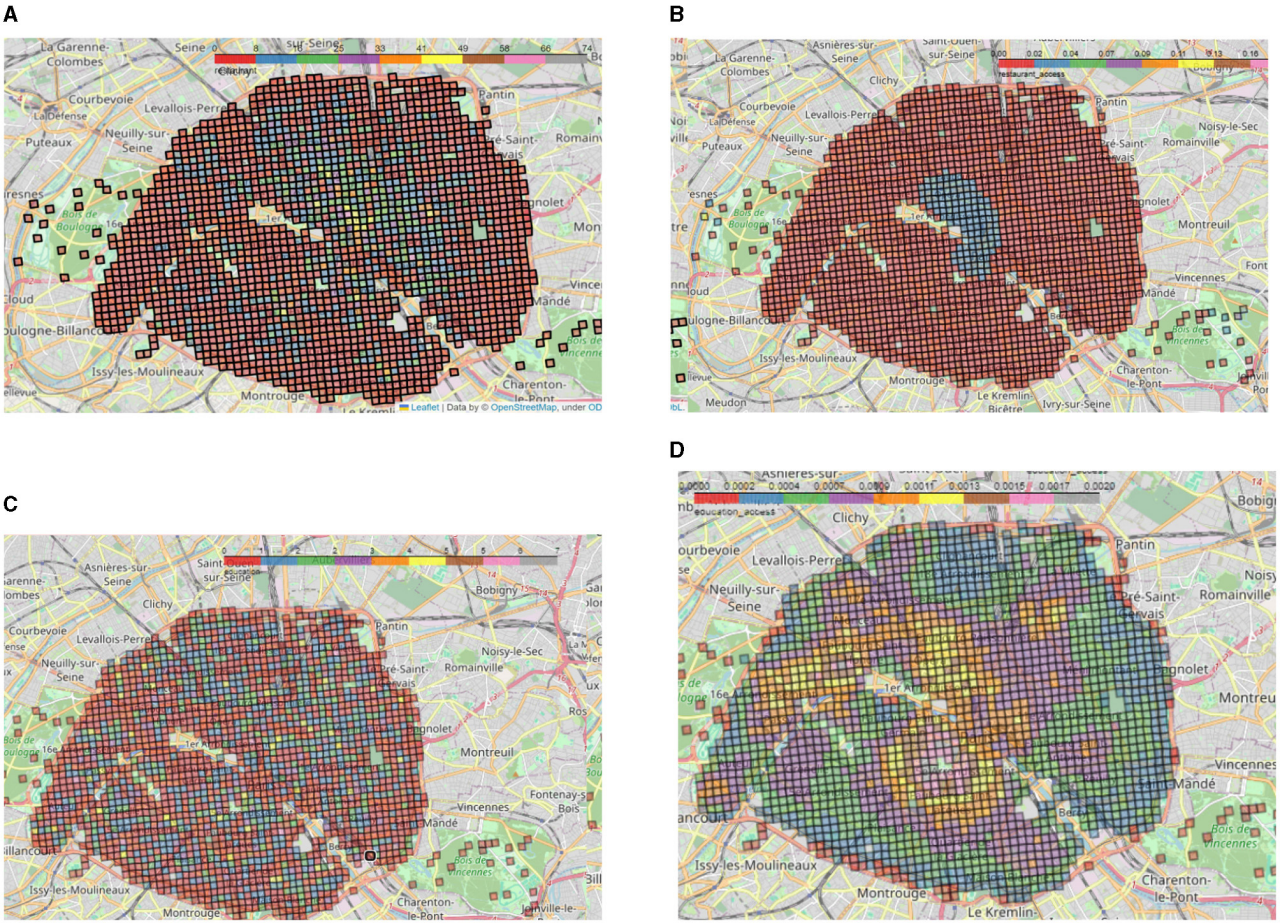
**(A)** Number of restaurants in Paris aggregated on the INSPIRE grid. Red squares of the grid depict 0–8 restaurants while gray squares contain between 66 and 74 restaurants.. **(B)** 2*FSCA*_*i*_(*p*) score of restaurants in Paris on the INSPIRE grid calculated for each of the 200 m × 200 m cells *i* and the amenity category *p* =*restaurant*. Red squares show very low accessibility (0 − 0.02) with up to 0.2 for gray squares. **(C)** Number of schools in Paris aggregated on the INSPIRE grid. Red squares of the grid contain no school at all while gray squares contain seven schools. **(D)** 2*FSCA*_*i*_(*p*) score of schools in Paris on the INSPIRE grid calculated for each of the 200 m × 200 m cells *i* and the amenity category *p* =*school*.

For each category (established on Section 2.1.3) or for each sub-type of amenities (*OSM* tags), we count the total number of items in each square in a grid (square of 200*m*×200*m*), which represents the supply *S*_*j*_ as defined in this section. This grid—named INSPIRE—comes from the *Filosofi* data set previously presented. We obtain, for instance, maps like in Figure 2A for restaurants or Figure 2C for schools in Paris. Then, for each category, we compute the 2SFCA score as shown in Figures 2B, D with, here, the number of restaurants and schools per square as the supply (and like for all computations, the number of inhabitants per square as the demand), respectively.

The figures depicting the number of restaurants and schools in Paris (Figures 2A, C) and the corresponding accessibility scores on the grid (Figures 2B, D) clearly show the importance of taking into account not only the total number but also accessibility measures of amenities. While the number of restaurants shows a strong trend toward the city center of Paris (*arrondissement* 1–4), the schools seem to be relatively well distributed among the city (Figure 2A). However, for schools (Figure 2D) the accessibility score clearly shows a concentration in the Passy district located in the South-West of Paris (16th), in the 5th/6th *arrondissements* (below the Seine) and in the 2nd/9th *arrondissements* (above the Seine, near the Opéra district). On top of the accessibility score for each category of amenities, we also calculate the accessibility score for housing and social housing thanks to the data provided by the *Filosofi* dataset.

In more detail, we note by *j* a square representing a supplier, while we denote by *i* a square consuming services (each square is both supplier and consumer). We also denote by *k* the index for the squares in the 1-km zone around *j*. We call *D*_*k*_ the demand (measured in inhabitants) in the square *k*, *S*_*j*_(*p*) the supply in the square *j*, measured in number of amenities of category *p*, *W*_*kj*_ the permeability coefficient or distance weight function of demand from *k* to go to square *j* as defined in Equation 1, and ℙ_*kj*_ the probability that inhabitants in square *k* visit square *j* (e.g., ℙ_*kj*_∝*W*_*kj*_).

**Figure 3:**
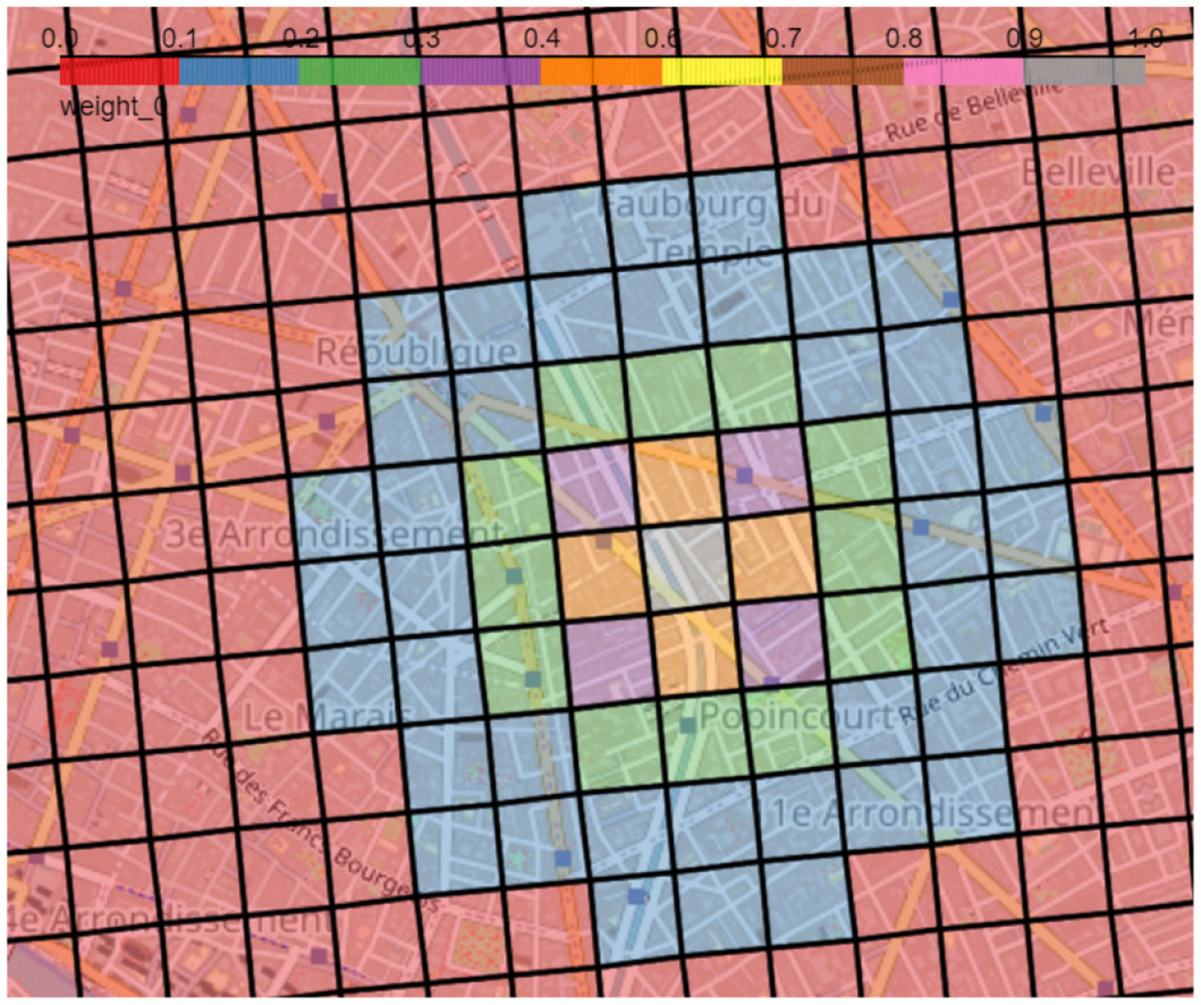
Weights associated to each square for the gray square in the center calculated as given in Equation 1, hence they are inversely proportional to the squared distance between the two geographical units. Red squares depict a weight between 0 and 0.1 and gray squares values between 0.9 and 1, while the sequence of colors in between shows intervals of 0.1 increasingly.

Here, we choose the following distance weight function, with *d*_*kl*_ the distance between *k* and *l*, being inversely proportional to the squared distance between two geographical units, also depicted in Figure 3:


(1) $$\begin{array}{l} W_{kl}=\frac{1}{d_{kl}^{2}}\text{, with:}d_{kl}\leq 1000m \end{array}$$

The distance weights are calculated using the PySAL library (Rey and Anselin, 2009) using the *DistanceBand* method, a continuous weight corresponding to the distance from the POI. We only consider a radius of 1*km* around the POI in order to look at amenities reachable within 15 min of walking as described above. Hence, we only calculate the distance weights within a distance of 1, 000*m*.

We can define the aggregated demand 𝔻_*j*_ received by the square *j*.


(2) $$\begin{array}{l} {𝔻}_{j}=\underset{k}{\sum }{\mathbb{P}}_{kj}D_{k} \end{array}$$

From that aggregated demand taking into account the zone around square *j*, we can compute the supply per inhabitant and amenity category *p* by taking their ratio *R*_*j*_(*p*).


(3) $$\begin{array}{l} R_{j}\left(p\right)=\frac{S_{j}}{{𝔻}_{j}}=\frac{S_{j}\left(p\right)}{\sum_{k}{\mathbb{P}}_{kj}D_{k}} \end{array}$$

And lastly, the accessibility indicator for the square *i* counted in number of services accessible in a one-kilometer radius per inhabitants, also denoted by 2*SFCA*_*i*_ score :


(4) $$\begin{array}{l} 2SFCA_{i}\left(p\right)=\underset{j}{\sum }{\mathbb{P}}_{ij}R_{j}\left(p\right) \end{array}$$

**Figure 4:**
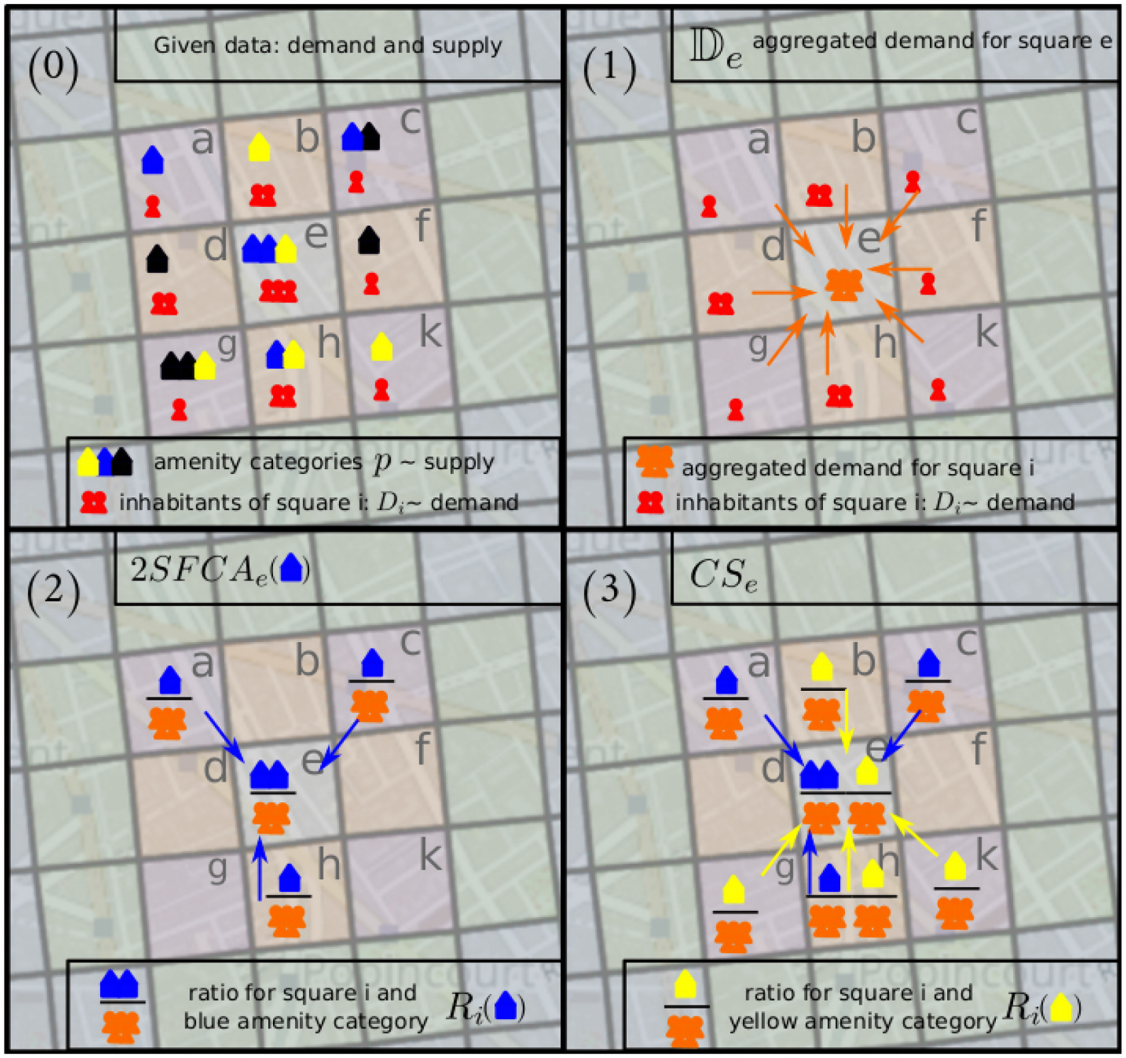
The calculation of the *CS*_*i*_, the aggregated 2SFCA score, is a stepwise process. (0) For each square in the grid, the demand and supply are given as the number of its inhabitants and the number of its amenities per category, respectively. (1) For each square, the aggregated demand is calculated based on inhabitants of all squares within a radius of 1 km. Here, the aggregated demand for square *e*, 𝔻_*e*_ is calculated. (2) In order to calculate the 2SFCA score for a square and an amenity category, the ratio of supply and aggregated demand is calculated for each square inside the 1 km radius. With square *e* as an example, we calculate 2*SFCA*_*e*_(*p*) with *p* being the blue amenity category. (3) For the calculation of the aggregated 2SFCA score for a specific square *i*, *CS*_*i*_, we combine all amenity categories, here in this example, we combine 2*SFCA*_*e*_(*p*) and 2*SFCA*_*e*_(*q*) with *p* being the blue amenity category and *q* being the yellow one.

As depicted in Figure 4, the aggregated accessibility score *CS*_*i*_ as shown in Equation 6 is calculated by the following step-wise process given the demand and supply for each square as measured in the number of inhabitants and the number of its amenities per category, respectively [Figure 4 (0)].

1. The aggregated demand 𝔻_*j*_ of a square *j* (Equation 2) is calculated for each square *j* [Figure 4 (1)];
2. The 2*SFCA*_*i*_(*p*) score (Equation 4) is calculated for a square *i* and amenity category *p* using the ratio *R*_*i*_(*p*) of supply for category *p* and the previously calculated aggregated demand for square *i* (Equation 3) and the probabilities ℙ_*ji*_ that people from square *j* visit square *i* [Figure 4 (2)];
3. The aggregated accessibility score *CS*_*i*_ (Equation 6, see following subsection) for a square *i* is calculated by taking into account all amenity categories for all squares in a radius of 1 km around square *i* as well as the amenity weight (Equation 5) and the min-max normalization given in Equation 7 [Figure 4 (3)].

#### 2.2.2 A basic measure of aggregated accessibility score

Knap et al. (2023) derived the amenity weights from the trip distributions calculated based on travel patterns in the their data. Our data sets did not allow for such calculations. To make up for this shortcoming, we have put forward the following hypothesis: the rarer a type of amenity, the more important it is. Schools or hospitals are less numerous than restaurants, because their capacity and importance is greater. We therefore calculate, for each type of amenity (i.e., for each category), the ratio between the number of items for the category *N*_*p*_ on the total number of amenities *N* and give an amenity weight opposite to this frequency.


(5) $$\begin{array}{l} w_{p}=\frac{N_{p}}{N}\text{where }p\text{ describes the categories established in}\\ \text{ Section 2.1.3.} \end{array}$$

The idea is that a scarcer amenity has greater importance. This amenity weight shows the inverse probability of finding an amenity of a certain category within all amenities in the area. Nevertheless, this hypothesis tends to overestimate certain amenities, such as museums, which are less numerous than some other categories but not necessarily more important to individuals.

This gives the aggregated 2*SFCA*_*i*_ for cell *i*, denoted by *CS*_*i*_:


(6) $$\begin{array}{l} CS_{i}=\overset{P}{\underset{p=1}{\sum }}\left(1-w_{p}\right)\times X_{i,p} \end{array}$$

where


(7) $$\begin{array}{l} X_{i,p}=\frac{2SFCA_{i}\left(p\right)-\min_{j}2SFCA_{j}\left(p\right)}{\max_{j}2SFCA_{j}\left(p\right)-\min_{j}2SFCA_{j}\left(p\right)} \end{array}$$

is the min-max normalization of the accessibility score 2*SFCA*_*i*_(*p*) of cell *i* for amenity of type *p* ∈ *P*.

#### 2.2.3 Regression

Regression analysis produces econometric results for Paris and the *Petite Couronne* that are comparable among different cities or regions. To do so, we set up a methodology as close as possible to the one introduced by Knap et al. (2023) for Utrecht.

The *spatial weight matrix*^11^ makes it possible to account for the geographical relationships and influences that exist between the different units in the database. There are two types of weights: contiguity weight and distance-based. Given the characteristic of Paris and our database (each unit is a square) we use only the first type. Three popular types of contiguity weights are called bishop, rook or queen weight as they take into account the adjacent squares of a grid depending on the figure's possible moves on a chess board (Loonis, 2018). As our dependent variables are demographic and social and in a highly urbanized context, the queen contiguity weight is justified based on the handbook (Loonis, 2018) stating that “the neighborhood in the sense of contiguity is often used to study demographic and social data, in which it may be more important to be on either side of an administrative boundary than to be located at a certain distance from one another.”

One way of checking whether a particular weight is relevant to a particular phenomenon is to calculate the Local Moran Index (LISA; Li et al., 2007). This indicator of spatial auto-correlation makes it possible to check whether a phenomenon is distributed randomly or, on the contrary, according to the spatial interactions between each unit. If it is close to 1 (resp. −1), there is a perfect spatial auto-correlation (resp. dispersion). We use here the local Moran Index in order to see where differences or outliers are located instead of receiving a single value from the global index which represents a summary of the relationships on the map.^12^

After calculating the local Moran index for our model on the accessibility score with queen weight for Paris, we find an index equal to 0.95 and a *p*-value equal to 0.01. This means that there is a strong spatial auto-correlation and that the hypothesis of a random distribution of the aggregate accessibility indicator (*H*_0_ : *random distribution*) can be rejected at the 95% threshold which confirms our choice for the queen weight. As for the tests performed on Paris, we obtain for the *Petite Couronne* a Moran index that is very close to 1 (0.97) and a very low *p*-value (0.001) which leads us to choose a queen weight for these regressions.

For our regression analysis, we use a model called *Spatial AutoRegressive with additional AutoRegressive error structure* (SARAR; Kelejian and Prucha, 1988; Anselin and Florax, 1995). We chose the model as the best fit for our purposes among several possibilities, for more information we refer to the Supplementary material. To implement our model we use the *spreg* library which computes this model using the generalized method of moments (GMM).^13^

To select our model we perform a Lagrange-Multiplier (LM) test which successively tests for the presence of spatial lag (robust and non-robust) (*H*_0_ : *p* = 0), the presence of spatial error (robust and non-robust) (*H*_0_ : λ = 0) and the joint presence of spatial error and spatial lag (*H*_0_ : *p* = λ = 0) (Anselin, 1988). As stated by Anselin et al. (1996), the LM test is a “simple diagnostic test for spatial dependence.” Here, λ corresponds to the spatial correlation effect of errors (spatial autocorrelation) and ρ is the endogenous interaction effect (spatial autoregressive). Moreover, the *p*-values obtained during the Lagrange-Multiplier tests are very close to 0, which confirms our choice of the SARAR model as the appropriate econometric model, see also Table 1.

**Table 1:** Results of the LM test for the aggregated 2SFCA score, *CS*_*i*_.

**LM test**	***p*-value**
LM error	0.00
LM lag	0.00
Robust LM error	0.00
Robust LM lag	0.00
LM SARMA	0.00

We have chosen to take into account heteroskedasticity to avoid errors in the significance of the coefficients. As in statistics, heteroskedasticity occurs when the variance of the residuals depends on the value of the variable of interest, i.e., the variance of the residuals decreases with the variable of interest. For example, as we analyze accessibility to amenities, we have to take into account that a person with a higher monthly income has a larger choice and thus, larger variability, of amenities than a person with a low income.

The results are presented in Table 3. Please see Section 2 in the Supplementary material for more details.

We run the regression of our aggregated 2SFCA on different socio-economic variables based on *Filosofi* data. The variables we work with are summarized in Table 2 below. We do not have some of the variables used in Knap et al. (2023), notably percentage of people receiving unemployment benefits, percentage of migrants, and distance to the nearest transport.

**Table 2:** Description of the variables used in the regression model.

**Variables**	**Description**	**Units**
%_soc.minimum	Percentage of households living below the social minimum threshold	%
%_≥_65	Percentage of individuals over 65 years of age	%
%_ ≤ _17	Percentage of individuals under 17 years of age	%
%_ ≤ _*bat*_45	Percentage of dwellings built before 1945	%
%_≥_*bat*_90	Percentage of dwellings built after 1990	%
%_residences	Percentage of collective residences	%
Mean_income	Mean yearly income per person (sum of winsorized^*^ living standards/number of inhabitants)	euro/person
Density	Residents per *km*^2^ (number of inhabitants in the square/(0.2 km*0.2 km)	pop/*km*^2^

^*^Winsorization is a statistical technique used to treat the extreme values of a distribution. In a given unit, an individual's standard of living is reduced to the 95th percentile of the unit distribution if her standard of living is higher than this threshold. Conversely, her standard of living is reduced to the 5th percentile of the unit distribution if her standard of living is below this threshold. If the standard of living is between these two thresholds, no treatment is carried out.

#### 2.2.4 MiniBatchKMeans clustering

Complementary to the regression analysis, we applied hierarchical clustering being an unsupervised method to create a typology of neighborhoods based on the accessibility measurements. The idea is to cluster on the different categories' accessibility as defined earlier, added to the accessibility of housing and social housing, to then define the main functions of different neighborhoods. To conclude, we correlate those functions with the socio-demographic characteristics of the population that inhabits the found clusters.

We carry out a clustering on accessibility measures on the aggregated categories we defined first (and not on the whole big dimensional space, for computation time reasons). We use MiniBatchKMeans clustering (Sculley, 2010). To choose the number of clusters, we carry out an elbow method with the distortion score as shown on Supplementary Figure 1: it suggests taking five clusters. See Section 3 in the Supplementary material for more details. MiniBatchKMeans is preferable because it is faster and also because it does not aggregate continuous squares such as AgglomerativeClustering (Nielsen, 2016). This particular fact allows to identify similar neighborhoods in different cities. The clustering has been done on around 2,000 squares for Paris and around 14,000 squares for *Petite Couronne*.

## 3 Results

Following our stepwise analysis as described in Section 2.2, we first describe the basic composition of 10 Parisian neighborhoods. Then we display the *CS*_*i*_ score (aggregated 2*SFCA*_*i*_ score) applied on the INSPIRE grid for Paris and the *Petite Couronne*, including and excluding the city of Paris itself. We depict the *CS*_*i*_ score on the grid. Here, we want to point out that the *CS*_*i*_ score is only to be used within the same figure and its values cannot be used to compare different settings. Hence, the colors are used to depict different values or intervals of values but are not generally comparable. We then present regression and clustering analyzes underlining the large differences between the city of Paris and its suburbs.

### 3.1 Basic descriptive statistics

To set the stage for our analysis, we first produce statistics to analyze the composition of a handful of districts in Paris. For this purpose, we arbitrarily choose a dozen of places, such as monuments or metro stations, around which the social life of the neighborhood is known to be organized. In each case, we limit the neighborhood to a perimeter of 1 km of the pedestrian network around the chosen location. In each neighborhood, we count the services for each of the categories defined above. We chose the POIs as depicted in Figure 1B in order to show the inhomogeneity inside the Parisian districts. Even tough the POIs are closely located to each other or even in the same *arrondissement*, the composition of their 1 km surroundings is quite different, for example when comparing Porte de la Chapelle ( b in Figure 1B) and Sacré Coeur ( d in Figure 1B), both in the 18th *arrondissement* in the North of Paris.

**Figure 5:**
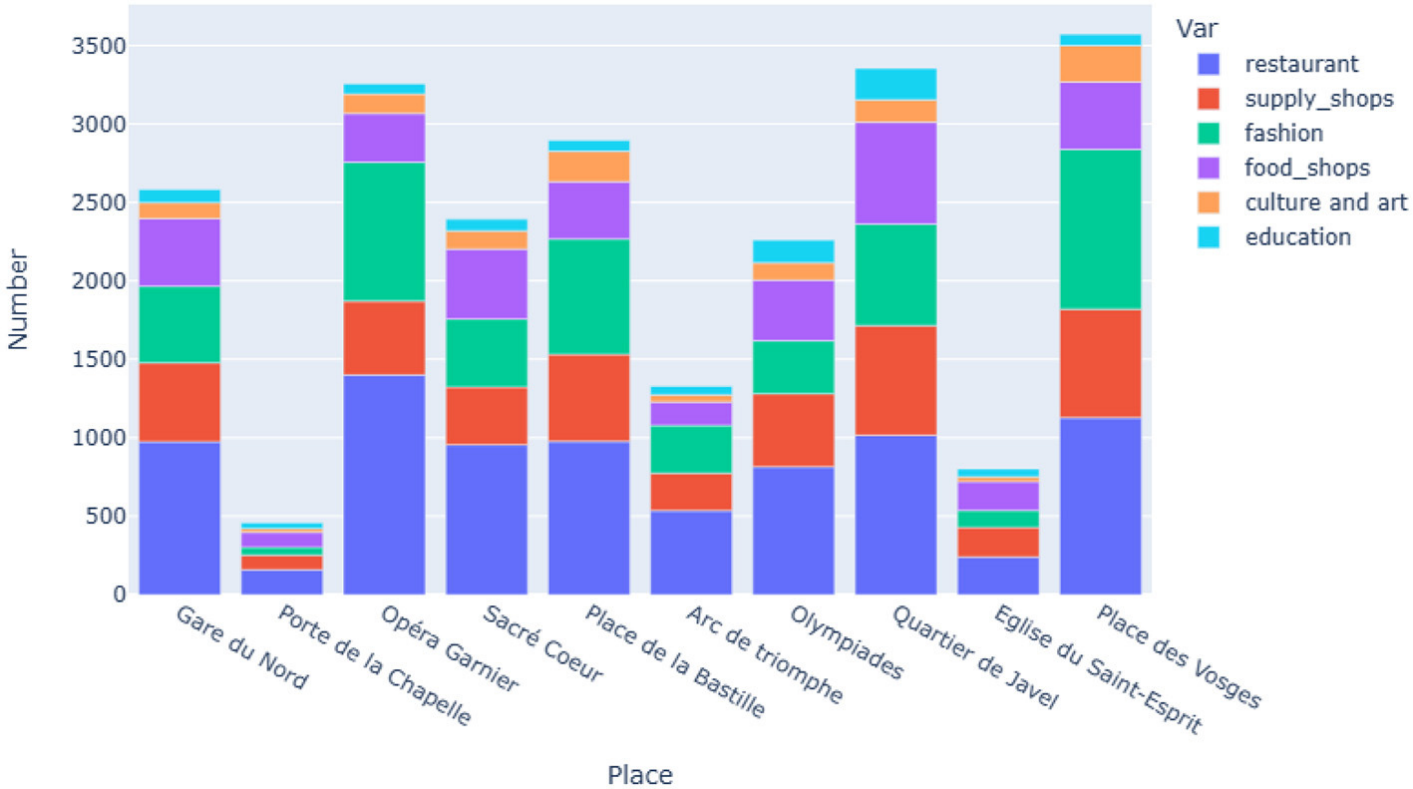
Comparison of the composition of the 1 km-surroundings of 10 Parisian POIs. Their locations are depicted in Figure 1B. Colors refer to the six different categories of services listed above, see Section 2.1.3. Figures 6A, B show the same data for the districts Opéra Garnier and Quartier de Javel on a percentage scale to underline the differences.

Figure 5 highlights disparities between neighborhoods in terms of the concentration of activities regarding the six different categories of services listed in Section 2.1.3. In particular, the most outlying neighborhoods, such as Porte de la Chapelle (18th *arrondissement*, b in Figure 1B) or Église du Saint-Esprit (Daumesnil district, 12th *arrondissement*, i in Figure 1B), which are also relatively poor neighborhoods (particularly Porte de la Chapelle), are those containing the fewest amenities.

The 18th *arrondissement* of Paris is marked by major inequalities: its northern part, where Porte de la Chapelle ( b in Figure 1B) is located, is home to some of Paris' most socio-economically disadvantaged populations. In contrast, the Butte Montmartre with Sacré Coeur (in the southern part of the 18th *arrondissement*, d in Figure 1B) is a major tourist attraction and home to a privileged population.

**Figure 6:**
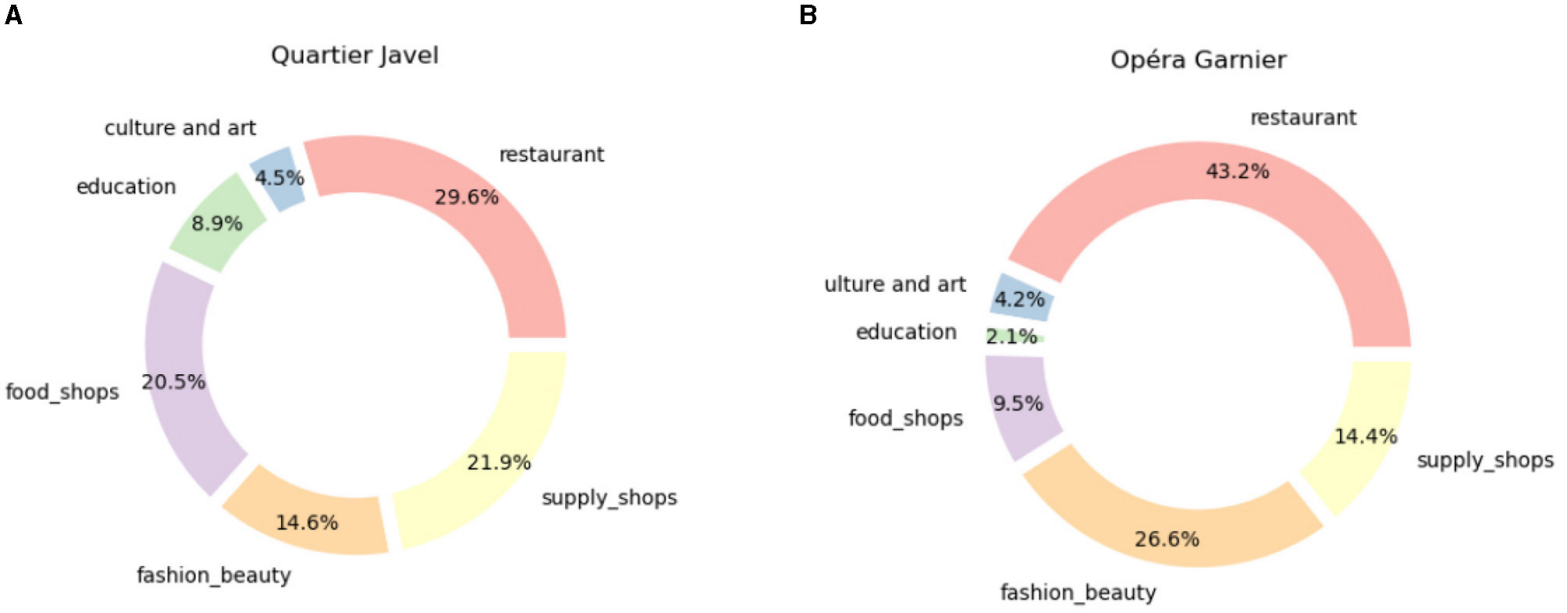
**(A)** Composition of the 1 km surrounding of Javel POI, corresponding to POI h in Figure 1B and the bar for Quartier de Javel in Figure 5. **(B)** Composition of the 1 km surrounding of Opera POI corresponding to POI c in Figure 1B and the bar for Opéra Garnier in Figure 5.

On the contrary, the districts around Place des Vosges (4th *arrondissement*, k in Figure 1B) and Opéra Garnier (9th *arrondissement*, c in Figure 1B), which are more central and more affluent, have a much higher number of amenities and also show significantly higher amounts in the categories of restaurants and fashion. Other rather outlying districts such as Javel (15th *arrondissement*, h in Figure 1B) and Olympiades district (13th *arrondissement*, g in Figure 1B) have a high number of amenities, especially in the category of education typical of residential areas. Thus, if the number of services is similar in the two cases, their composition distinguishes a touristic district (Opéra Garnier, Figure 6B) from a more residential one (Javel district, Figure 6A). More than 50% of the Javel district is composed of daily services (food shops, supply shops, and education), compared to 26% around Opéra Garnier. Instead, the latter has almost 70% of luxury services (restaurants, fashion), compared to 44% in the former.

### 3.2 Aggregated 2SFCA

**Figure 7:**
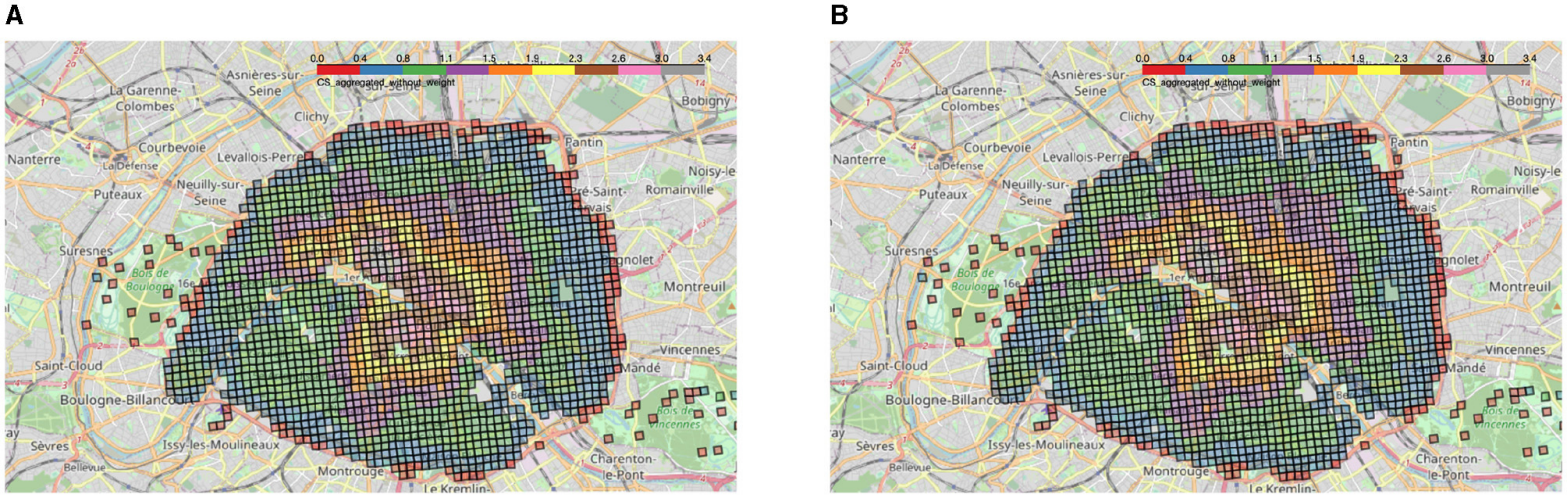
**(A)** Aggregated 2SFCA without amenity weights for Paris. **(B)** Aggregated 2SFCA with amenity weights for Paris.

We created the aggregated accessibility score with and without the amenity weights *w*_*p*_ (Figures 7A, B). As can be seen on these two maps, there is little difference between the two methods, but as expected, the accessibility score with amenity weights tends to emphasize the presence of schools. As a matter of fact, this is particularly true in the Passy district located in the South-West of Paris in the 16th *arrondissement*, which has a stronger score with weights because of the presence of many high schools (Saint-Jean de Passy, Saint-Louis de Gonzague, etc.) as shown in Figure 2D. In the following, we chose to keep the weighted score : although there are some biases, it seemed more appropriate as it provides us with an approximation of the importance of each amenity. Moreover, ignoring the amenity weights would risk invisibilizing less numerous amenities: restaurants in excess could invisibilize the importance of rarer amenities such as schools.

In both cases, a concentric circle gradient in the aggregated access to amenities can be observed, starting from three main centers : Opéra Garnier (9th arr), Les Halles (1st arr), and the Latin Quarter (5th/6th arr). This confirms the descriptive statistics for the Opéra district conducted earlier (Figure 6B). For the Latin Quarter, this can be explained by the strong presence of both museums and universities/secondary schools. As for the Halles district, there is a large shopping center as well as a strong economic activity in the surroundings.

**Figure 8:**
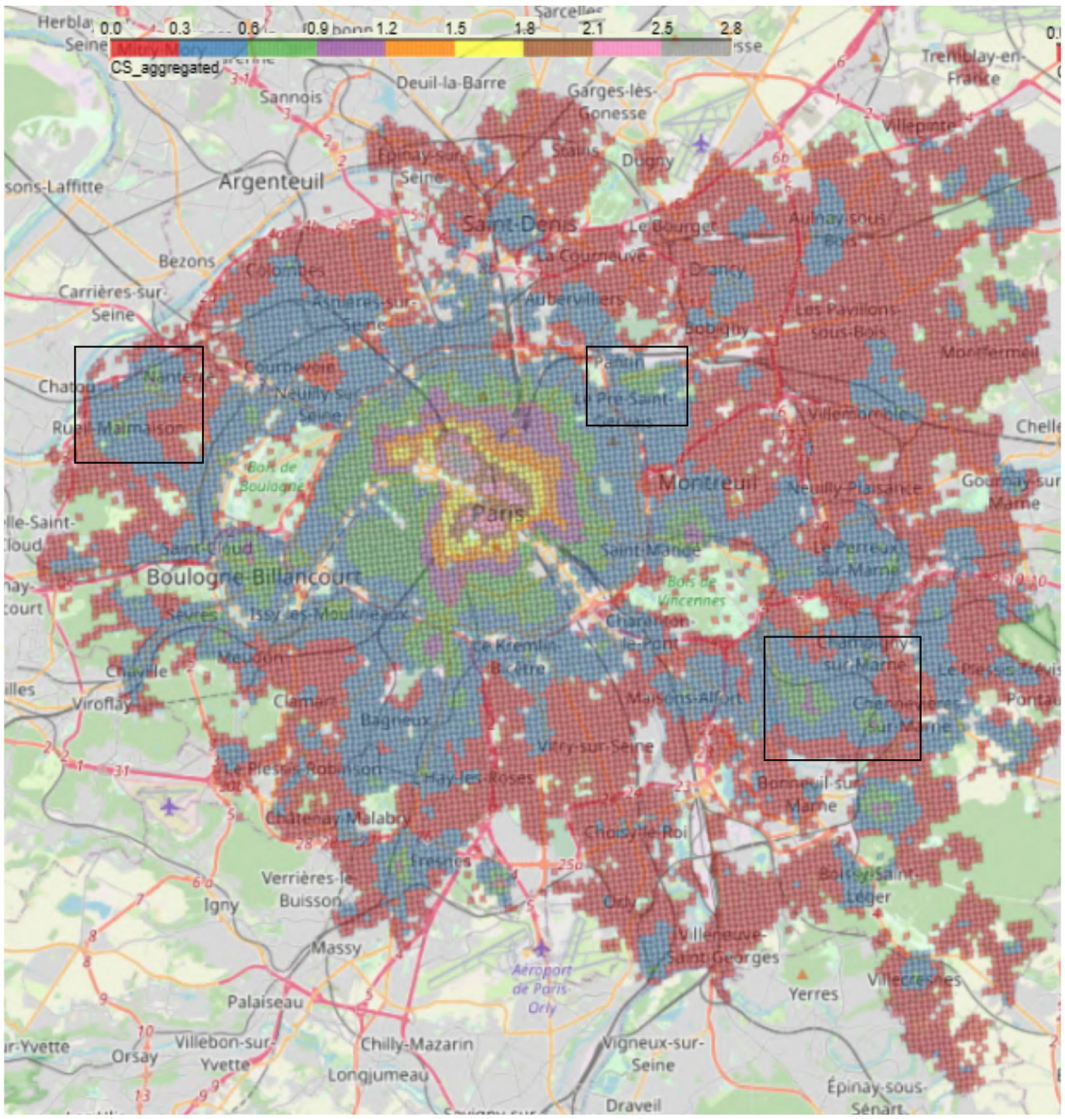
Aggregated 2SFCA (*CS*_*i*_) score for the complete *Petite Couronne* including Paris. The rectangles correspond to the zoomed versions, the left rectangle shows Figure 9A, the middle rectangle Figure 9B and the right one shows Figure 9C. The colors in the legend correspond to different values of the aggregated 2SFCA score, whereas red shows the lowest and gray the highest accessibility scores.

In Figure 8, that represents the weighted aggregate score of accessibility to amenities for the entire *Petite Couronne*, we can also notice a concentric circle that starts in Paris and extends over the entire *Petite Couronne*. Services are highly concentrated within Paris and the nearby suburbs are less endowed with amenities. This is consistent with INSEE results (Trevien, 2017) on inequality of access between households living in urban vis-à-vis suburban areas.

**Figure 9:**
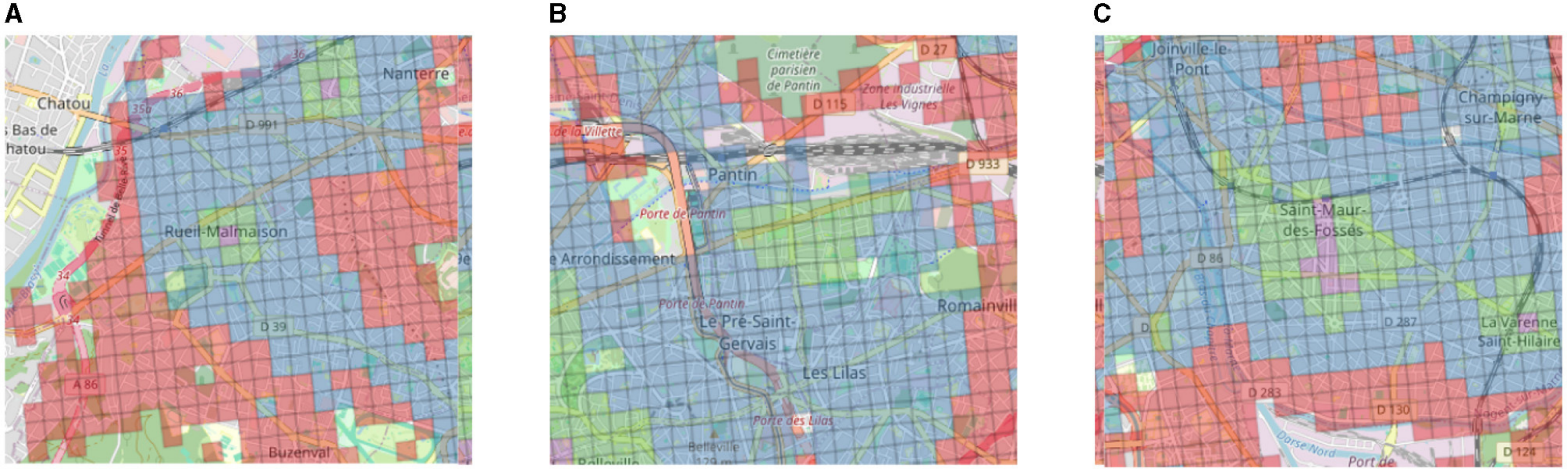
**(A)** Zoom of the aggregated 2SFCA for the town of **Rueil Malmaison** located in the West, close to the *Bois de Boulogne* (16th arrondissement) and left rectangle in Figure 8. **(B)** Zoom of the aggregated 2SFCA for the area of the shopping mall **Paddock Paris** opened in 2019 and located North-East of Paris (close to the 19th arr.) and middle rectangle in Figure 8. **(C)** Zoom of the aggregated 2SFCA for the town of **Saint Maur** located South-East of the *Bois de Vincennes* (12th arrondissement) and right rectangle in Figure 8.

However, even in the suburbs, there is once again intense activity in the center of the towns at the expense of the edges: this is the case, for example, for Rueil-Malmaison (Figure 9A) and Saint-Maur (Figure 9C). Additionally, shopping center projects in the suburbs lead to the creation of areas with a especially high density of amenities. One example is the shopping center *Paddock Paris*,^14^ North-East of Paris near Pantin, which has 17,000 stores and was opened in 2019. As depicted in Figure 9B, we can see an elevated *CS*_*i*_ score in comparison to its surroundings.

**Figure 10:**
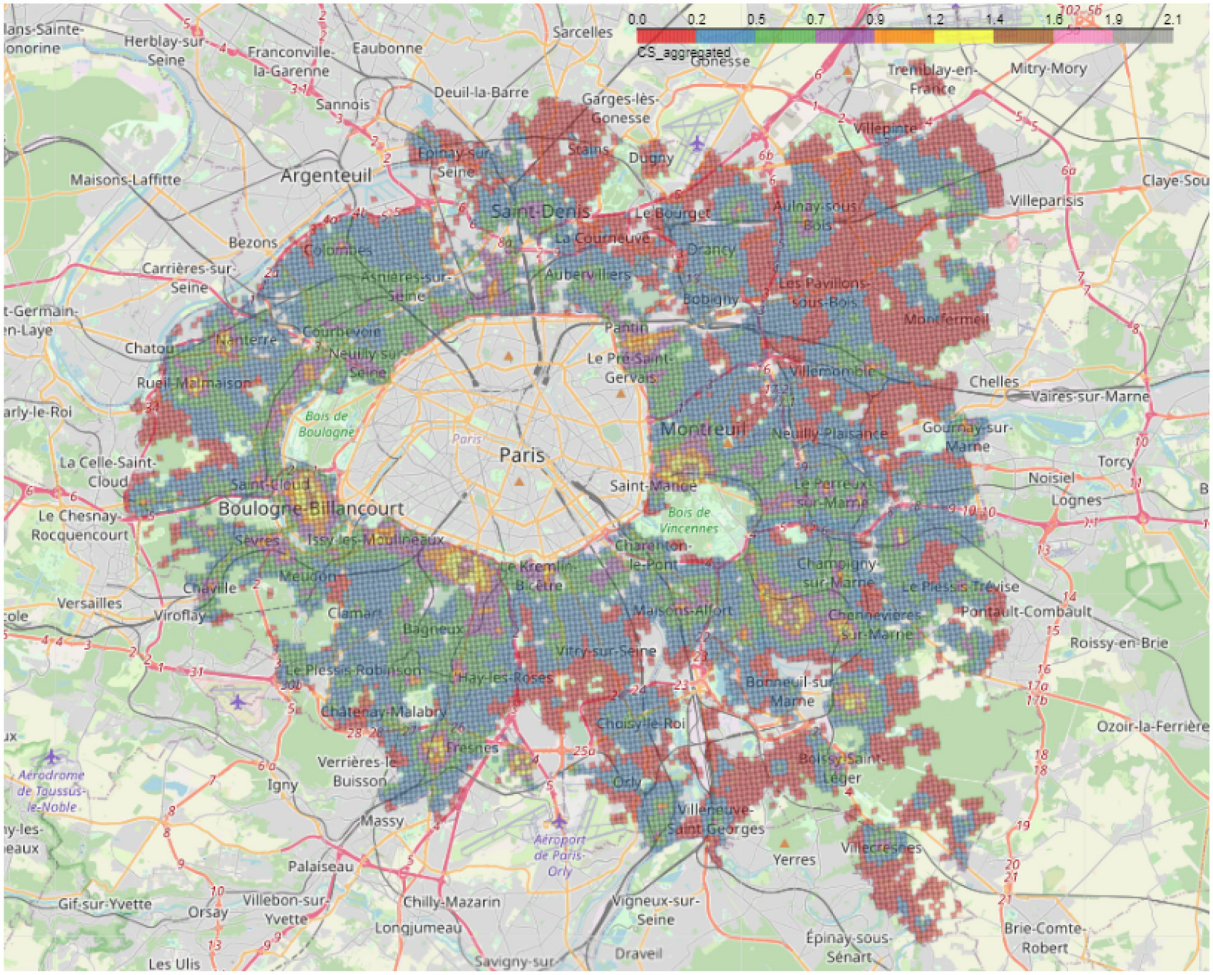
Aggregated 2SFCA score for the *Petite Couronne* without Paris.

It is well-known that Paris is a city marked by strong segregation, particularly between the inner and outer suburbs (Clerval, 2016). Hence, we expect to see a strongly biased distribution of accessibility scores regarding the whole region of *Petite Couronne* including the city of Paris. We rerun the calculation of the scores excluding Paris. By plotting the aggregated accessibility score on the Petite Couronne excluding Paris (depicted in Figure 10), we find the dynamic centers within the cities that have already been identified: the city centers of Neuilly-sur-Seine, Issy-les-Moulineaux, Saint-Mandé, Boulogne-Billancourt, Rueil-Malmaison, Nanterre offer a wide range of services and activities as they achieve a high accessibility score, see Figure 1A. However, their scores increase significantly, while the scores of deprived areas (e.g., Châtenay-Malabry in the South or La Courneuve in the North, points **K** and **L** in Figure 1A, respectively) remain more or less the same. Thus, excluding Paris shows a clearer differentiation among the suburbs as it unveils the wealthy towns and the poorer areas outside Paris.

### 3.3 Regression

#### 3.3.1 Regression analysis for Paris

The results of the regression analysis for Paris, as listed in Table 3 (left), indicate that the higher the percentage of the population below 17 years old or above 65 years old, the lower the *CS*_*i*_ score. This outcome may seem a little surprising but is consistent with the results found in Utrecht (Knap et al., 2023). One explanation could be that families and oldest households prefer to live in less “dynamic” areas like the 15th *arrondissement*. These neighborhoods are quieter during the day and at night, i.e., there are fewer bars.

**Table 3:** Spatial regression results for Paris and the *Petite Couronne*.

	**Paris**					***Petite Couronne***				
**Variables**	β	**Std. error**	***Z*** **-statistic**	***P*** **-value**	**Sig**	β	**Std. error**	***Z*** **-statistic**	***P*** **-value**	***Sig***
Constant	0.22310	0.03373	6.61346	0.00000	***	–0.00430	0.0047322	–0.90808	0.36384	
%_soc.minimum	–0.00144	0.00076	–1.90452	0.05684	*	–0.00043	0.0001574	–2.75186	0.00593	***
%_≥65	–0.00070	0.00032	–2.17647	0.02952	**	–0.00001	0.0000846	–0.10111	0.91946	
%_ ≤ 17	–0.00271	0.00039	–7.01182	0.00000	***	–0.00033	0.0000933	–3.50424	0.00046	***
%_ ≤ _*bat*_45	0.00010	0.00007	1.38763	0.16525		0.00020	0.0000245	8.36205	0.00000	***
%_≥_*bat*_90	0.00013	0.00010	1.34830	0.17756		0.00005	0.0000168	3.04687	0.00231	***
%_residences	–0.00169	0.00028	–6.15153	0.00000	***	0.00024	0.0000254	9.28002	0.00000	***
Mean_income	–0.00000	0.00000	–1.52188	0.12804		–0.00000	0.0000001	–0.25953	0.79523	
Density	0.00000	0.00000	9.14481	0.00000	***	0.00000	0.0000000	11.80775	0.00000	***
**Model fit**:										
Pseudo *R*^2^	0.9879					0.9834				
Spatial Pseudo *R*^2^	0.0056					0.4363				

***P ≤ 0.01.

**P ≤ 0.05.

*P ≤ 0.1.

Results are obtained using the SARAR model. The columns in the table show values for the coefficients of the regression analysis, the standard error, the *z*-Statistic and the corresponding *p*-value. The last columns indicate the significance based on the *p*-value for Paris and the *Petite Couronne*, respectively.

However, the percentage of households living below the social minimum, the percentage of buildings built before 1945 and the average income are not significant at the 5% level regarding the results of the regression analysis. This indicates that, in Paris, accessibility to essential amenities cannot be explained by the poverty rate of a neighborhood and hence, economic criteria are not useful to predict the level of accessibility to essential amenities in Paris. With regard to the buildings built before 1945, this can be explained by the presence of almost all buildings dating from the nineteenth century in Paris. This also explains the high *p*-value of the percentage of buildings built after 1990. Because of the homogeneity of density in Paris, there is no relationship between our metric and the density of the neighborhood, in contrast to what the INSEE study of 2017 seemed to indicate throughout France as a whole (Trevien, 2017).

#### 3.3.2 Regression analysis for the *Petite Couronne* including Paris

We have seen that in Paris, young people under 17 have relatively less access to services than adults under 65 *ceteris paribus*. But while socio-economic-criteria do not shape inequality in accessibility to services, for the *Petite Couronne* we observe that the coefficient of *%_soc.minimum* is significant and negative: the more households living below the minimum social threshold, the lower the accessibility score, as listed in Table 1 (right). If Paris is a relatively homogeneous city in terms of socio-economic level, then the poorest households will tend to move to the outskirts of Paris where the cost of living is lower. Performing the analysis on the *Petite Couronne* and not only on Paris therefore allows us to take into account socio-economic inequalities that are not noticeable within Paris.

In addition, the coefficients on the percentage of buildings built before 1945 and after 1990 are significant: geographic units containing more buildings built before 1945 and after 1990 have a better accessibility score to services than units containing buildings built between 1945 and 1990. This could be a consequence of the urban policies implemented during the *Trente Glorieuses* period,^15^ aiming to respond to the post-war housing crisis: in 1953 with the Courant plan^16^ these policies, based on the principle of zoning,^17^ resulted in the construction of large residential areas on the outskirts of Paris far from shops and services. Moreover, the positive relationship between the percentage of buildings built after 1990 and the *CS*_*i*_ metric could be due to the presence of this type of building in freshly renovated neighborhoods and more office spaces. Eventually, we observe that geographic units containing more buildings built after 1990 have a relatively poorer accessibility score than units containing pre-war buildings: this could reflect the disparities between Paris and its suburbs observed in Figure 8, as the majority of Parisian buildings were built under Haussmann.^18^ Paris is a fairly old city whose architecture is fairly well-protected by law: most of the Haussmannian districts are protected, and there has been little destruction since the 1980's. For more details, see Clerval (2016). It can also be noted that the coefficients of *%_residences* and *density* are significant: the more houses rather than apartment blocks are located in an area, the lower the accessibility score. In addition, the more densely populated an area, the higher its accessibility score.

### 3.4 Clustering

**Figure 11:**
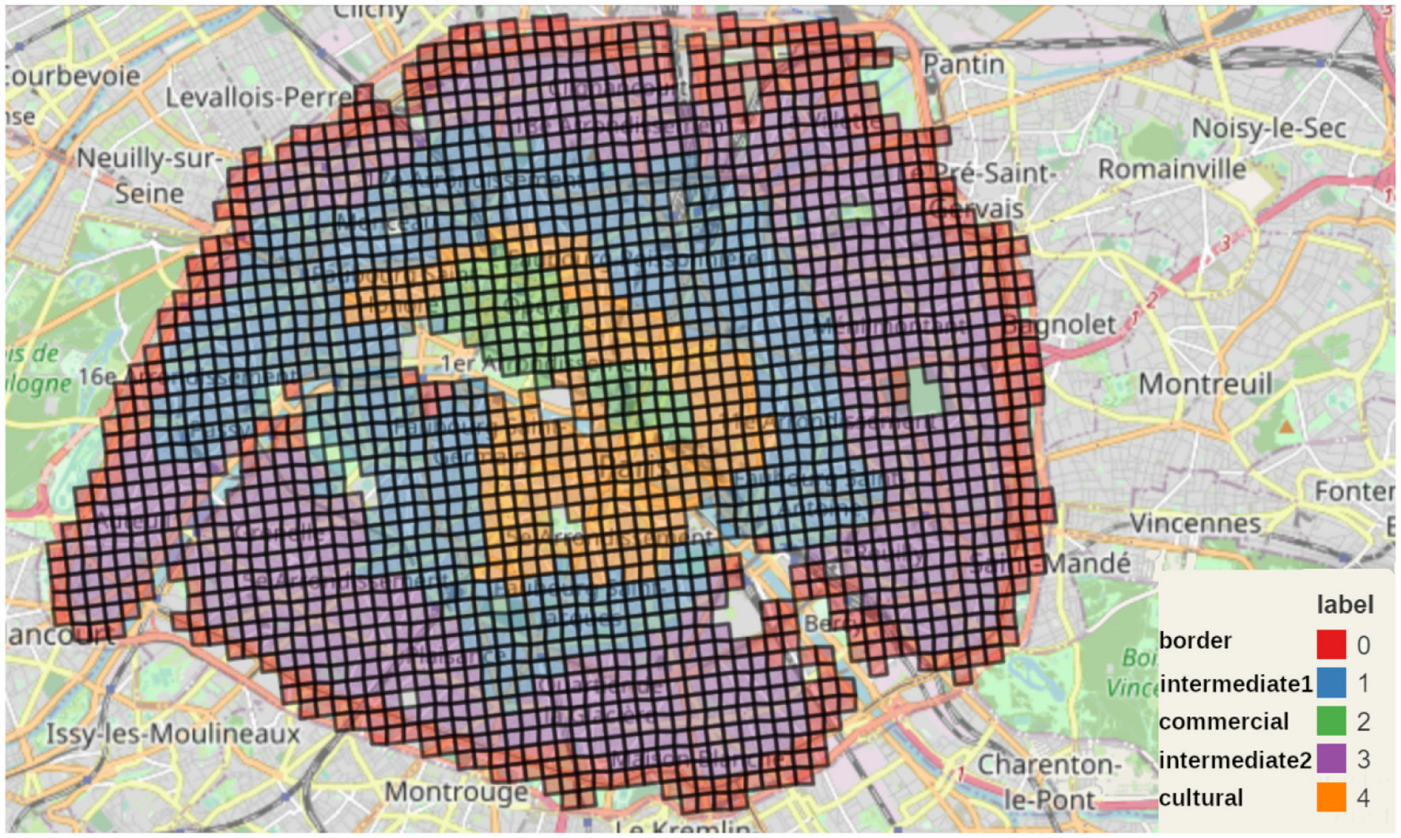
Map of the five MiniBatchKMeans clusters for Paris.

**Figure 12:**
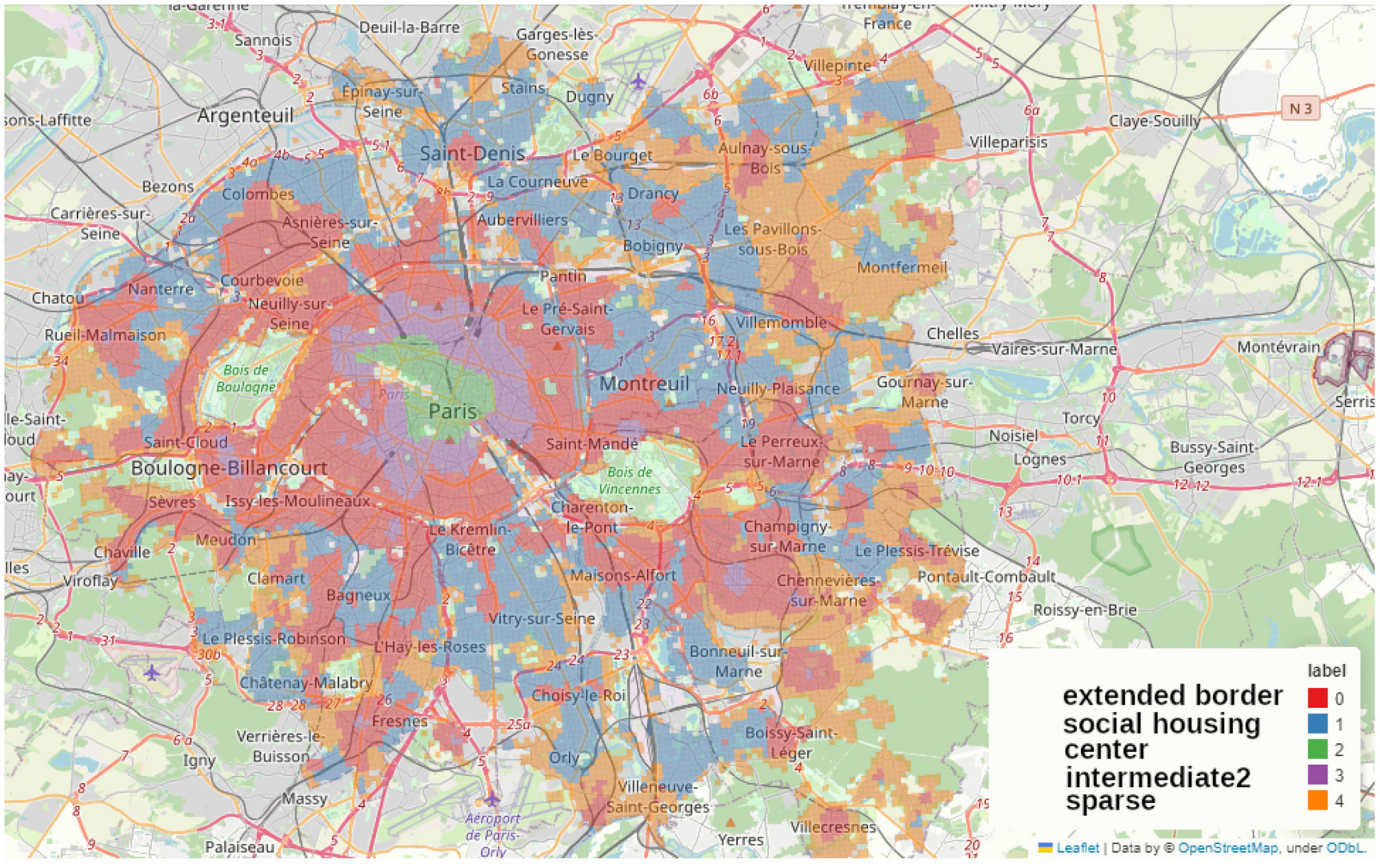
Map of the clusters made using MiniBatchKMeans method on the accessibility measurements at the scale of the whole *Petite Couronne* including Paris, we shortly term this map *PCP* when referring to its clusters.

**Figure 13:**
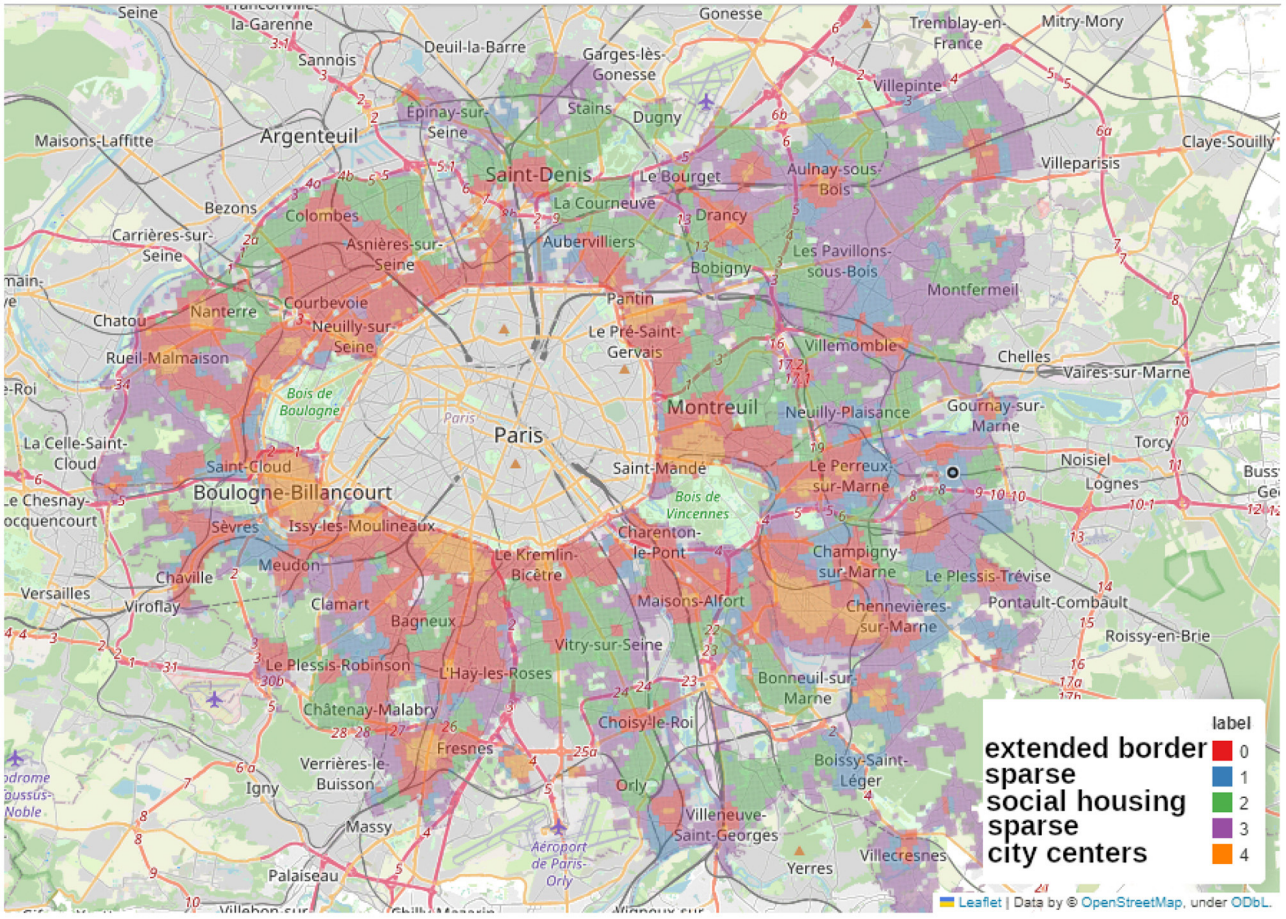
Map of the clusters made using MiniBatchKMeans method on the accessibility measurements at the scale of the whole *Petite Couronne* excluding Paris, we shortly call this map *PC* when describing its clusters.

We applied the MiniBatchKMeans clustering to Paris alone (Figure 11), to the complete *Petite Couronne* including Paris (Figure 12) as well as without Paris (Figure 13). We chose to use 5 clusters as suggested by the elbow method as described in the Methods Section 2.2.4 and Supplementary Figure 1.

In the current section, we describe the features of the five clusters obtained by the MiniBatchKMeans clustering and comment on similarities and differences. Therefore, we assigned names to the clusters in all of the figures. Similar or equal names describe corresponding clusters. The figures depict Paris alone (Figure 11) which we call P, the complete *Petite Couronne* including Paris (Figure 12) here called PCP and the complete *Petite Couronne* without Paris, called PC.

In case we detected differences of the distribution of the aggregated accessibility score among different clusters, we ran a *t*-test for each of them in order to confirm statistical significance. Each of them were significant at the 95% level.

#### 3.4.1 Clustering analysis for Paris

Regarding the clusters obtained for Paris as depicted in Figure 11, we can clearly differentiate various areas. The green cluster (*P-commercial*) corresponds to the commercial function previously identified. Indeed, with a closer look, we see that it matches *les Halles* (1st arr.), well-known for its shopping center, and the *Opéra* neighborhood (9th arr.), well-known for its shops. The orange cluster (*P-cultural*) seems more centered on cultural amenities compared to the green one, that's why it includes *Le Marais* (3rd arr.) and *Saint Germain-des-Prés* (6th arr.), two neighborhoods with plenty of art galleries, and the *Quartier Latin* (5th and 6th arr.), which is not only an educational center but also a cultural one, as shown by (Apur, 2023). Those impressions are quantitatively confirmed as shown on Supplementary Figure 2. Here, we can see that the clusters *P-commercial* and *P-cultural* are standing out for the overall amenities' accessibility, though cluster *P-commercial* is more inclined toward fashion, food shops, restaurants and supply shops whereas cluster *P-cultural* has higher accessibility to culture and arts and to education.

The external cluster is being divided into three clusters : the red (*P-border*) one which corresponds to the border, the blue (*P-intermediate1*) one and the violet (*P-intermediate2*) one. The cluster *P-border* stands out. Its geographical location is on the border of Paris, and it is the poorest in terms of accessibility apart from social housing. Multiple explanations can be given to that cluster. At first, it could be an artifact due to the arbitrary limit of the data to Paris itself. However, historically the first real development of social housing in Paris - the so-called *Habitat Bon Marché*, literally cheap habitat—occurred precisely in those areas at the edge of Paris, as shown in a study by the *Atelier Parisien d'Urbanisme* (APUR; (Arènes et al., 2019)). This historical fact (Stèbè, 2022) could explain the importance of social housing on the border of Paris (excluding the edges of the *Bois de Boulogne*, 16th arr). Also, Paris and its suburbs have been separated by the *périphérique*, an important ring road around the city. So the lack of amenities can also be the physical effect of that ring road. However, this cluster can also be found on some edges of the Seine and on some edges of the *cimetière du Père Lachaise* (20th arr) in the North. To conclude, the *P-border* cluster is probably the mixed consequence of being on the edges (of Paris, of the Seine, of parks) and the history of Parisian planning where the first social housing have been in place on those edges. This history triggers socio-economic consequences, comparatively isolating poorer communities from the rest of amenities.

The comparison between clusters *P-intermediate1* and *P-intermediate2* is easier. Apart from social housing and housing, *P-intermediate1* has better accessibility in every aspect, even though it is still behind *P-commercial* and *P-cultural*. Regarding the socio-demographic description of each cluster, as shown in the different violin plots of Supplementary Figure 3, the *P-border* cluster is clearly the poorest cluster by looking at the percentage of poor households and the mean standard of living, followed by *P-intermediate2*. This relative poverty is probably partly due to the fact that the red cluster includes more vulnerable families, like single-parent and very large households. We can also see that this cluster is home to mostly renters. Social housing, renting, presence of socially difficult situations and poverty therefore co-occur.

The cluster *P-intermediate2* seems to host a similar amount of poor people, but the mean income is lower. This cluster is probably home to a small middle class. We can also look at the fact that it is hosting the denser squares, which is corroborated by the geography of the cluster : the 13th, the 15th, the 19th, the 20th are the most populated *arrondissements*. By contrast, *P-commercial* and *P-cultural* are the least dense and the richest clusters. In particular, *P-cultural* one is hosting the richest square in all Paris. Barplots describing the distribution of the aggregated accessibility score and of the socio-economic variables are shown in Supplementary Figures 2, 3.

#### 3.4.2 Clustering on *Petite Couronne* including Paris

We perform the same clustering as for the city of Paris for the whole region of the *Petite Couronne*, at first including Paris as depicted in Figure 12 and then without Paris in order to see if the data on Paris introduces a strong bias, see Figure 13.

Regarding the complete *Petite Couronne* with Paris (Figure 12), we quickly find back the same center (in green, *PCP-center*) as in the cluster analysis of Paris (*P-commercial* and *P-cultural*), showing that it does stand out, no other center seems to match it. Some other city centers can be identified in the violet (*PCP-intermediate2*) cluster, which is the cluster of Paris “middle ground” neighborhoods, *P-intermediate2*. Globally, the same city centers are standing out as in the map obtained with the aggregated accessibility score and highlighted in Figures 9A, C. They are for instance Reuil-Malmaison, Saint-Maur, Montrouge, Boulogne-Billancourt, see also Figure 1A. The *department* of Seine-Saint-Denis in the north of the *Petite Couronne* seems to be the only territory without any well-equipped city center. In this part of the region, there is the largest part of less equipped clusters (orange, *PCP-sparse*) and red (*PCP-extended-border*). The *PCP-sparse* cluster is the least equipped of all clusters (even in terms of housing and social housing). This cluster is probably representative of a suburb (*banlieue pavillonaire*) with low density of housing and amenities accessible by walking. On the contrary, the blue (*PCP-social-housing*) and red (*PCP-extended-border*) clusters are denser, with more housing and social housing, and are more compatible with the 15-min city because more amenities are accessible by walking. Barplots describing the distribution of the aggregated accessibility score and of the socio-economic variables are shown in Supplementary Figures 4, 5.

#### 3.4.3 Clustering on *Petite Couronne* excluding Paris

Clustering on the *Petite Couronne* excluding Paris essentially finds the clusters predicted by the aggregated accessibility score as depicted in Figure 13. The orange cluster (*PC-city-centers*) stands out from the others: access to amenities is particularly high, but there's no particular socio-economic profile. However, it seems to match the city centers of the suburban cities.

The green cluster (*PC-social-housing*) stands out particularly strongly from the others: it has a younger, poorer population. Access to social housing is particularly high, and access to amenities is relatively lower than in the other clusters. Both clusters called *social-housing* correspond to each other, the green cluster (*PC-social-housing*) in Figure 13 matches the blue cluster (*PCP-social-housing*) in the previous clustering analysis, depicted in Figure 12. Barplots describing the distribution of the aggregated accessibility score and of the socio-economic variables are shown in the Supplementary Figures 6, 7.

## 4 Discussion

Combining open map data from a large participatory project and geo-localized socio-economic data from official statistics, the above analysis has endeavored to assess the extent to which Paris is a 15-min city, where residents can access essential amenities such as restaurants and shops with a 15-min walk or bike ride. We show that it is not sufficient to calculate the number of amenities within a certain area but it is essential to develop measurements for accessibility of amenities as a means to analyze the current situation of a city and its inhabitants. The accessibility score then shows which areas need to be improved or remodeled in order to achieve walkable neighborhoods where inhabitants can reach essential amenities within a walk of around 15 min. Additionally, we do not only demonstrate the inequalities within Paris and its suburbs but also the access to amenities such as culture and art or luxury shops. With the clustering, we aim to demonstrate the different areas regarding accessibility measures in correspondance with socio-economic data. Our results match with results and data from related studies. We now summarize our main results.

### 4.1 Summary of main results

We have developed a statistical model using different types of POIs to analyze the composition of a big city like Paris and its surroundings. After defining categories of amenities, we have applied the 2SFCA aggregate accessibility measure, which takes into account supply and demand for each type of service.

Our analyzes are based on the following hypotheses, regarding the accessibility of an amenity:

- The further away the services are, the less likely they are to be visited and the less accessible they are;
- the higher the demand for an amenity, the less accessible it is;
- the lower the supply for an amenity, the less accessible it is.

The analysis sheds light on a strong dichotomy of access within Paris: on the one hand, there is a lively commercial area in the heart of the city and on the other hand, more residential neighborhoods on the outskirts, with less access to services. Composed of the surroundings of Les Halles (1st arr.), the Latin Quarter (5th and 6th arr.), the Opéra Garnier area (9th arr.) and the Champs Élysées (8th arr.), the bustling center fulfills two distinct functions made visible by two distinct clusters for the center of Paris: while the activity located along the Champs Élysées, around the Opéra and les Halles (being located North of the Seine river, 1st, 8th and 9th *arrondissements*) is more inclined toward luxury shops, food shops, restaurants and supply shops, the Latin Quarter is focused on cultural and educational activities (located South of the Seine river, 5th and 6th arr.). The analysis also reveals inequalities in access to services correlated to socio-economic criteria: the poorest neighborhoods, which are located on the edge of the city, are the least endowed with amenities.

Regarding the disparities of access according to people's age as the youngest live in the neighborhoods with fewer services shows that families with underage children live mainly in less-endowed areas. Hence, one can conclude that Paris as a city is excessively expensive for families with children such that they are forced to live in the suburbs or move to less attractive areas.

Extending the analysis to the *Petite Couronne* has further illuminated the inequalities in access to services. We have shown that services are highly concentrated within Paris, to the detriment of its nearby peripheries. The same difference is observed within these suburban towns and cities, where accessibility is high in the center and low on the edges. Extension to the *Petite Couronne* highlights socio-economic inequalities in access to facilities that were less visible for Paris: households living below the minimum social threshold and those living in areas built between 1945 and 1990 have poor access to amenities. The cluster analysis establishes not only an opposition between bustling centers and edges but also a dichotomy between low accessibility areas; not obviously seen through the aggregated indicator: those sparse with low density of housing, and on contrary, those denser with an over representation of social housing.

To conclude, the overall cluster analysis allows us to better understand the composition of Paris at different levels of detail, highlighting differences in the type of amenities, past the aggregated accessibility.

Firstly, Paris is driven by a dichotomy between a really well-equipped center and the rest. Secondly, this center itself is composed of a commercial area on *Rive Droite* (right riverside, North of the Seine river), and an educational, cultural and artistic pole on *Rive Gauche* (left riverside, South of the Seine river). These two centers are surrounded by well-equipped, but less specialized neighborhoods. The rest of Paris can be decomposed into three big spaces: a border (itself decomposed into two parts), residential and popular spaces (for instance the 15th, 13th, 19th, and 20th *arrondissements)* which are slightly better equipped than the border and have globally the same number of social housing, and finally a rich space which includes the 7th, 8th, 9th, and 16th *arrondissements* and consists of the most well-equipped neighborhoods outside the very center.

One of the most determinant variables is the accessibility of social housing, which drives half of specificity of the border and the specificity of the popular neighborhoods. The demography seems also an important factor: the central clusters are characterized by their low percentage of minors, which corroborates what we found with the regression. People live and have children mostly outside the really well-equipped centers. These places of residence tend to be divided between those close to social housing, and those in sparse areas.

### 4.2 Key implications for further research

With the above analysis of the 15-min city concept in Paris and its suburbs based on a combination of mapping data and socio-economic variables, we have identified inequalities within Paris, between Paris and suburbs, and between different areas within suburbs. Although Paris is quite homogeneous in its composition, there is a huge gap in comparison to the rest of the *Petite Couronne*. However, people living in the suburbs take an important role in Parisian life as many of them are daily commuters, workers, and users who contribute to shaping the city. Numerous studies on the 15-min city concept take Paris as good example, however, with our results, we claim that any analyzes of 15-min or X-min city concepts should not be limited to the city itself but rather include the suburban regions into account when formulating policies and redesigning the city. Ignoring a city's suburbs means excluding many people from the analysis who play essentials roles for the functioning of the city even though they are not inhabitants of the city itself.

However, the analysis carried out in this project has some limitations and possibilities of future extensions that we point out in what follows.

### 4.3 Data limitations

First, the use of *OSM* data may have constrained our analyzes. Although its geographical quality is attested by a review by IGN officers, who evaluated *OSM* data in France as relatively good (Girres and Touya, 2010), the database is filled in by anonymous volunteers, which can lead to errors and heterogeneous information. Because we lack some information, the list of categories established in Section 2.1.3 may be incomplete: for example, we do not have access to medical care, access to green spaces and sports infrastructures or the possibility of finding a job near home. Moreover, *OSM* data neither provide quality and price information on the amenities, nor measures of their importance (as may be captured for example by number of visits). Without this information, we should take with caution the above regression results which present economic criteria as not significant, suggesting an image of Paris as a rather egalitarian city in terms of accessibility.

New iterations of the *Filosofi* data set, not yet released to the public, will include more socio-economic data. Future extensions of this study may thus extend the set of categories to medical care, green spaces and sport infrastructures. Additional data sets can be included in order to complete missing information, such as *Foursquare*.^19^

We are aware of the fact that our accessibility score measures the demand in terms of the population while the supply is measured in terms of the number of amenities of the category in question which does not include the different capacities of single amenities. Measuring the capacity of an amenity is a difficult task as already its definition is unclear. Taking a bakery as an example, we face the following questions: is it the number of people at any time? the maximum of this last number? the total amount of items sold in one day? It is even harder for a hospital : is the equivalent of full time jobs working in the hospital? is the number of beds? It is sufficiently hard that INSEE publishes accessibility numbers only for liberal medical entities and not for hospitals. Hence, we conclude that adding a (arbitrary) measure of capacity to our analysis will not add a meaningful value to our results.

Lastly, while our measurement is representative of the spatial and demographic composition of amenities in Paris, we do not take into account the demand by non-inhabitants like tourists. Thus, we may be probably overestimating the availability of for instance the central restaurants. The same analysis with a second data source (like the SIRENE database,^20^ or the APUR collection^21^) could be a way to check this bias.

### 4.4 Enriching the accessibility score

Future research can enrich the 2SFCA method in three ways. First, we can incorporate *S*_*j*_ (resp. *R*_*j*_, the supply) in the probability that inhabitants from square *i* visit *j*. It is called 3SFCA (resp. fixed point SFCA method) (Wan et al., 2012). In each case, the idea is that people take into account not only distance but also supply in square *j*, in line with the idea that supply creates (partly, at least) its own demand. The second improvement is to adjust the demand function in regard to the socio-demographic composition of each square. For instance, we can expect that young people “consume” more schools than older people. These adjustments can be made, for instance, using the mean consumption of each demographic group at the national scale as used in INSEE studies (Trevien, 2017; Cazaubiel and Cohen, 2020). As we have the demographic pyramid of each square, we can construct for each category a consumption indicator based on national accounts data. Lastly, the distance could be computed using the distance on the road network rather than the distance as crow flies, although the analysis would require much bigger computing resources (due to the complexity of the graph exploration times the complexity of the number of square).

### 4.5 Inclusion of public transportation

To better capture the dynamics that make Paris an accessible city, it would probably helpful to re-compute the 15-min area by also taking into account public transport. This will change the accessibility of many neighborhoods along metro lines. Hence, including public transportation will change (i) the accessibility scores as access to public transport will be included as another category of services and (ii) the 15-min radius of an inhabitant as the usage of public transportation will increase distances. Data for public transportation in French cities is available via INSEE^22^ or RATP^23^ providing data for Paris and its suburbs.

### 4.6 Going back in time

In order to detect areas of gentrification or other impactful changes, it will be necessary to look at data sets from different time intervals. In this way, it will be possible to study the evolution of POIs over several years and see the change with, i.e., the installation of new districts and shopping centers. As described above, the shopping center Paddock Paris is such an example. It was opened in 2019 and, we can see an elevated *CS*_*i*_ score in comparison to its surroundings in Figure 9B.

A limitation here will be the availability of data. OSM is a relatively young project that started in 2004^24^ and hence, poses a restriction on the analysis of earlier states of cities.

In sum, future extensions of this research may include an extension of the 2SFCA score taking into account further categories of services as well as additional data sets.

## Data availability statement

Python scripts, notebooks and further information on software tools and libraries used are openly available in our github repository (https://github.com/LeoMaurice/BATO-MOUCHE-Stat-App). Further inquiries can be directed to the corresponding author.

## Author contributions

M-OT: Data curation, Methodology, Software, Visualization, Writing – original draft, Writing – review & editing. SG: Data curation, Methodology, Software, Visualization, Writing – original draft, Writing – review & editing. LM: Data curation, Methodology, Software, Visualization, Writing – original draft, Writing – review & editing. PT: Conceptualization, Funding acquisition, Investigation, Methodology, Project administration, Supervision, Validation, Writing – original draft, Writing – review & editing. SB: Conceptualization, Funding acquisition, Investigation, Methodology, Project administration, Supervision, Validation, Visualization, Writing – original draft, Writing - review & editing.

## Footnotes

^1^https://www.bbc.com/worklife/article/20201214-how-15-minute-cities-will-change-the-way-we-socialise and https://www.lejournaldugrandparis.fr/la-ville-du-1-4-dheure-au-coeur-du-programme-danne-hidalgo/

^2^ https://prizeforcities.org/project/15-minute-city

^3^ https://www.inegalites.fr/Le-palmares-des-villes-francaises-les-plus-inegalitaires

^4^A Point-of-Interest (POI) is a term used in cartography to denote a place or location on a map that someone might find interesting or useful, for example a restaurant, a hotel, a tourist attraction, or even a more ordinary place like a school.

^5^INSEE is the French national institute of statistics and economic studies.

^6^In geography, a distance “as the crow flies” is a distance measured in a straight line.

^7^ https://www.insee.fr/fr/statistiques/4176290?sommaire=4176305#consulter

^8^ https://wiki.openstreetmap.org/wiki/FR:Key:shop

^9^ https://wiki.openstreetmap.org/wiki/FR:%C3%89l%C3%A9ments_cartographiques

^10^ https://www.itf-oecd.org/benchmarking-accessibility-cities

^11^ https://geographicdata.science/book/notebooks/04_spatial_weights.html

^12^ https://geographicdata.science/book/notebooks/07_local_autocorrelation.html##local-moran-s-i-i

^13^ https://pysal.org/spreg/generated/spreg.GM_Combo_Het.html#spreg.GM_Combo_Het

^14^ https://www.visitparisregion.com/fr/paddock-paris-est

^15^Les *Trente Glorieuses* was the 30-year period of economic growth in France between 1945 and 1975, corresponding to reconstruction after the end of the Second World War.

^16^In order to deal with the large demand of housing, the French government decided in 1953 for the *Courant plan*, allowing to quickly build large housing complexes https://francearchives.gouv.fr/fr/pages_histoire/39082.

^17^Zoning is a method of urban planning in which a municipality or other level of local government divides the land into zones, each of which serves a specific need.

^18^Haussmann was charged with the renovation of Paris and direct suburbs in the years between 1853 and 1870, removing old medieval buildings, constructing parks, avenues and squares. This was thought to improve the situation regarding the high population density, the spread of diseases, and traffic circulation. Hence, the street plan of Paris today has largely been designed by Haussmann. https://en.wikipedia.org/wiki/Haussmann%27s_renovation_of_Paris.

^19^ foursquare.com

^20^ https://www.sirene.fr/sirene/public/accueil

^21^ https://www.apur.org/fr

^22^ https://www.insee.fr/en/statistiques/series/102775080?INDICATEUR=2774981

^23^ https://data.ratp.fr/explore/?sort=modified

^24^ https://osmfoundation.org/wiki/Main_Page
