# Supplementary material for: City composition and accessibility statistics in and around Paris
Source: Front Big Data. 2024 Mar 1;7:1354007. doi: 10.3389/fdata.2024.1354007 (PMC10941645; doi:10.3389/fdata.2024.1354007)
Supplement: Supplementary file 1 [file Data_Sheet_1.PDF]

Supplementary Material

1 OSM KEYS

Chosen OSM keys are listed in Table S1.

Category	*OSM* key	*OSM* tags
**Restaurant**	amenities	bar, biergarten, cafe, fast_food, food_court, ice_cream, pub, restaurant
**Culture and art**	amenities	arts_centre, cinema, conference_centre, events_venue, library, music_school, planetarium, public_bookcase, studio, theatre, toy_library
	shops	anime, antiques, art, books, camera, collector, craft, frame, games, model, musical_instrument, music, photo, ticket, trophy, video, video_games
**Education**	amenities	college, kindergarten, school, university
**Food shops**	shops	alcohol, bakery, beverages, brewing_supplies, butcher, cheese, chocolate, coffee, confectionery, convenience, dairy, deli, farm, frozen_food, greengrocer, ice_cream, pasta, pastry, seafood, spices, tea, wine, water, supermarket
**Fashion and beauty**	shops	bag, boutique, beauty, clothes, cosmetics, erotic, fabric, fashion_accessories, hairdresser, hairdresser_supply, jewelry, leather, massage,perfumery, sewing, shoes, tailor, tattoo, watches, wool
**Supply shops**	shops	agrarian, appliance, atv, baby_goods, bathroom_furnishing, bed, bicycle, boat, bookmaker, candles, cannabis, car, caravan, car_parts, carpet, car_repair, charity, chemist, computer, copy-shop, curtain, department_store, do-it-yourself, doors, dry_cleaning, e-cigarette, electrical, electronics, energy, fireplace, fuel, fishing, flooring, florist, fuel, funeral_directors, furniture, garden_centre, garden_furniture, gas, general, gift, glaziery, golf, groundskeeping, hardware, health_food, hearing_aids, herbalist, hifi, household_linen, housewar, hunting, insurance, interior_decoration, jetski, kiosk, kitchen, laundry, lighting, locksmith, lottery, mall, medical_supply, military_surplus, mobile_phone, money_lender, motorcycle, newsagent, nutrition_supplements, optician, outdoor, outpost, paint, party, pawnbroker, pest_control, pet, pet_grooming, pyrotechnics, radiotechnics, religion, scuba_diving, security, ski, snowmobile, sports, stationery, storage_rental, swimming_pool, telecommunication, tiles, tobacco, toys, trade, trailer, travel_agency, tyres, vaccum_cleaner, weapons, window_blind

Table S1. *OSM* tags selected in each category

2 REGRESSION ANALYSIS

The regression analysis has been done to obtain econometric results for Paris and the *Petite Couronne* that are comparable among different cities or regions. To do so, we set up a methodology as close as possible to the one introduced by Knap et al. for Utrecht (Knap et al., 2023).

2.1 Spatial Weight Matrix

The spatial weight matrix makes it possible to account for the geographical relationships and influences that exist between the different units in the database. There are two types of weights: contiguity weight and distance-based[1]. Given the characteristic of Paris and our database (each unit is a square) we will only be interested in the first type. Three popular types of contiguity weights are called bishop, rook or queen weight as they take into account the adjacent squares of a grid depending on the figure's possible moves on a chess board.

One way of checking whether a particular type of weight is relevant to a particular phenomenon is to calculate the Local Moran Index (LISA) (Li et al., 2007). This indicator of spatial auto-correlation makes it possible to check whether a phenomenon is distributed randomly or, on the contrary, according to the spatial interactions between each unit. If it is close to 1 (resp. -1), there is a perfect spatial auto-correlation (resp. dispersion)[2].

2.2 The different spatial regression models

For econometric reasons, the Ordinary Least Square (OLS) model is not suitable for spatial data. Indeed, the spatial auto-correlation of the residuals (i.e. the dependence between nearby observations) violates the assumptions leading to a loss of OLS efficiency or biased estimators. There may be a spatial lag and/or an auto-correlation of errors (spatial auto-correlation or spatial error in the models) introducing an auto-regressive effect into the model. Therefore, we must rely on econometric models thought for spatial data.

Spatial error Model (SEM) : This model defined by Anselin Anselin (1988) and Lesage and Pace LeSage and Pace (2009), introduces a spatial lag in the error term.

$$Y_i = \alpha + X_i\beta + u_i \tag{S1}$$

$$u_i = \lambda u_{lag-i} + \epsilon_i$$

With X_i the independent variables, α the constant, $u_{lag-i} = \sum_{j\neq i} w_{i,j}u_j$, ϵ_i the error term and $w_{i,j}$ the spatial weight. The parameter λ represents the intensity of the interdependence between the u error terms.

Spatial lag Model (SAR or SLM) : This model developed by Anselin et BeraAnselin and Bera (1998), introduces a spatial lag ρ in the dependent variables.

$$Y_i = \alpha + \rho W Y_{lag-i} + X\beta + \epsilon_i \tag{S2}$$

[1] For more information on the spatial weight matrix, please refer to https://geographicdata.science/book/notebooks/04_spatial_weights.html

[2] For more information on the Moran Index, please refer to https://geographicdata.science/book/notebooks/07_local_autocorrelation.html#local-moran-s-i-i

With $Y_{lag-i} = \sum_{j \neq i} w_{i,j} Y_j$, W designs the spatial weight matrix. This model looks at the spatial interdependence with the other variables. The endogenous lag is calculated by running a TWO-SLS regression with the spatial lag of all explanatory variables as the instrument for the endogenous lag.

Spatial AutoRegressive with additional AutoRegressive error structure (SARAR)(Kelejian and Prucha, 1988; Anselin and Florax, 1995)[3]: This model defined by Kelejian and Prucha (1988), combines the two previous approaches [4].

$$Y = X\beta + \rho WY + \lambda u + \epsilon \tag{S3}$$

To implement our models we use the *spreg* library which computes this model (`https://pysal.org/spreg/generated/spreg.GM_Combo_Het.html#spreg.GM_Combo_Het`) using the generalized method of moments (GMM).

Model selection : To select our model we perform a Lagrange-Multiplier (LM) test which successively tests for the presence of spatial lag (robust and non-robust) ($H_0 : p = 0$), the presence of spatial error (robust and non-robust) ($H_0 : \lambda = 0$) and the joint presence of spatial error and spatial lag ($H_0 : p = \lambda = 0$).

2.3 Choice of independent variables

We run regressions of our aggregate 2SFCA on different socio-economic variables based on *Filosofi* data, similarly to the analysis in the article on the city of Utrecht Knap et al. (2023). The variables we work with are summarized in Table S2 below. Unlike the article, we do not have the percentage of people receiving unemployment benefits, nor the percentage of migrants, nor the distance to the nearest transport.

Variables	Description	Units
%_soc.minimum	Percentage of households living below the social minimum threshold	%
$\%\_ \geq \_65$	Percentage of individuals over 65 years of age	%
$\%\_ \leq \_17$	Percentage of individuals under 17 years of age	%
$\%\_ \leq \_bat\_45$	Percentage of dwellings built before 1945	%
$\%\_ \geq \_bat\_90$	Percentage of dwellings built after 1990	%
%_residences	Percentage of collective residences	%
mean_income	Mean yearly income per person (sum of living standards windorised/nb of inhabitants)	euro/person
density	Residents per km^2 (number of inhabitants in the square/(0.2km*0.2km)	pop/km^2

Table S2. Variables used in the regression model, the same table can be found in the main manusript.

Our model was built with a queen weight. As a matter of fact, after calculating the local Moran index for the queen weight on the accessibility score, we find an index equal to 0.95 and a p-value equal to 0.01. This means that there is a strong spatial auto-correlation and that the hypothesis of a random distribution of the aggregate accessibility indicator (H_0 : *random distribution*) can be rejected at the 95% threshold. Therefore, we can consider that the queen weight is to be taken into account for our regressions.

[3] It should be noted that there is also the Spatial Durbin Model (SDM) which also combines the two previous approaches but whose implementation is not available on *Python*. The Durbin model is the most widely used and is generally recommended.

[4] For more information on the SARAR model, see also `www.insee.fr/en/information/3635545`

After implementing the Lagrange-Multiplier test, we find that the adequate model for our two regressions is the SARAR model. We have chosen to take into account heteroskedasticity[5] to avoid errors in the significance of the coefficients. The results are presented in the main manuscript Table 2 (the β values correspond to those of model, Equation S3).

As for the tests performed on Paris, we obtain for the *Petite Couronne* a Moran index that is very close to 1 (0.97) and a very low p-value (0.001) which leads us to choose a queen weight for these regressions. Moreover, the p-values obtained during the Lagrange-Multiplier tests are very close to 0, so the appropriate econometric model is also the SARAR model. We also decide to take into account a possible heteroskedasticity.

3 CLUSTERING

We carry out a clustering on accessibility measures on the aggregated categories we defined first (and not on the whole big dimensional space, for computation time reasons). We use a MiniBatchKMeans (Sculley, 2010) with 5 clusters (suggested by the elbow method). MiniBatchKMeans is preferable because it is faster and also because it does not aggregate continuous squares such as AgglomerativeClustering (Nielsen and Nielsen, 2016). This particular fact allows to identify similar neighbourhoods in different cities. The clustering has been done on around 2 000 squares for Paris and around 14 000 squares for *Petite Couronne*.

However, to better understand the composition of Paris neighbourhoods, it is not satisfactory. To complete the analysis, we decide to do a MiniBatchKMeans clustering with more than two clusters. To choose the number of clusters, we carry out an elbow method with the distortion score as shown on Figure S1 : it suggests taking 5 clusters. To confirm that number of clusters, we calculate the silhouette score as shown on Figure S1. We encounter a slight problem : the silhouette score suggests taking two clusters. At first, it confirms what we find with the MeanShift method : Paris is driven by an opposition between a touristic, commercial centre and the others residential neighbourhoods. It is also possible that the silhouette score is being wronged by the fact that one of the cluster is an *"edge"* cluster, as it is described in the results section.

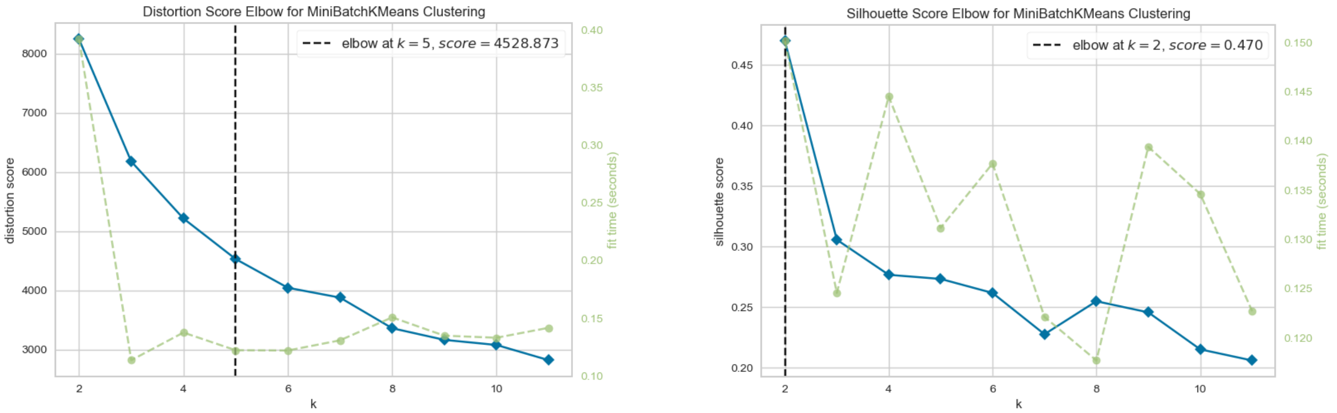

Figure S1. Elbow method for two scores (blue) for the clustering on the categories accessibility depicting the distortion score (left) and silhouette score (right). Green is the computation time.

[5] In statistics, heteroskedasticity occurs when the variance of the residuals depends on the value of the variable of interest. For example, the variance of the residuals decreases with the variable of interest.

3.1 Cluster details for Paris

Figures S2 and S3 depicting the distribution of the aggregated accessibility score among different clusters.

3.2 Cluster details for the *Petite Couronne* including Paris

Figures S4 and S5 depicting the distribution of the aggregated accessibility score among different clusters.

3.3 Cluster details for the *Petite Couronne* excluding Paris

Figures S6 and S7 depicting the distribution of the aggregated accessibility score among different clusters.

REFERENCES

Anselin, L. (1988). *Spatial Econometrics: Methods and Models*. Studies in Operational Regional Science (Springer Netherlands)

Anselin, L. and Bera, A. (1998). English (US)*Spatial Dependence in Linear Regression Models with an Introduction to Spatial Econometrics* (CRC Press). 237–290. doi:10.1201/9781482269901-36

Anselin, L. and Florax, R. J. (1995). Small sample properties of tests for spatial dependence in regression models: Some further results. In *New directions in spatial econometrics* (Springer). 21–74

Kelejian, H. H. and Prucha, I. R. (1988). A generalized spatial two-stage least squares procedure for estimating a spatial autoregressive model with autoregressive disturbances. *Journal of Real Estate Finance and Economics* 17:1

Knap, E., Ulak, M. B., Geurs, K. T., Mulders, A., and van der Drift, S. (2023). A composite X-minute city cycling accessibility metric and its role in assessing spatial and socioeconomic inequalities–A case study in Utrecht, the Netherlands. *Journal of Urban Mobility* 3, 100043

LeSage, J. and Pace, R. (2009). *Introduction to Spatial Econometrics Statistics*. Series:Statistics:TextbooksandMonographs (BocaRaton,Florida:CRCPress)

Li, H., Calder, C. A., and Cressie, N. (2007). Beyond moran's i: Testing for spatial dependence based on the spatial autoregressive model. *Geographical Analysis* 39, 357–375. doi:https://doi.org/10.1111/j.1538-4632.2007.00708.x

Nielsen, F. and Nielsen, F. (2016). Hierarchical clustering. *Introduction to HPC with MPI for Data Science* , 195–211

Sculley, D. (2010). Web-scale k-means clustering. In *Proceedings of the 19th international conference on World wide web*. 1177–1178

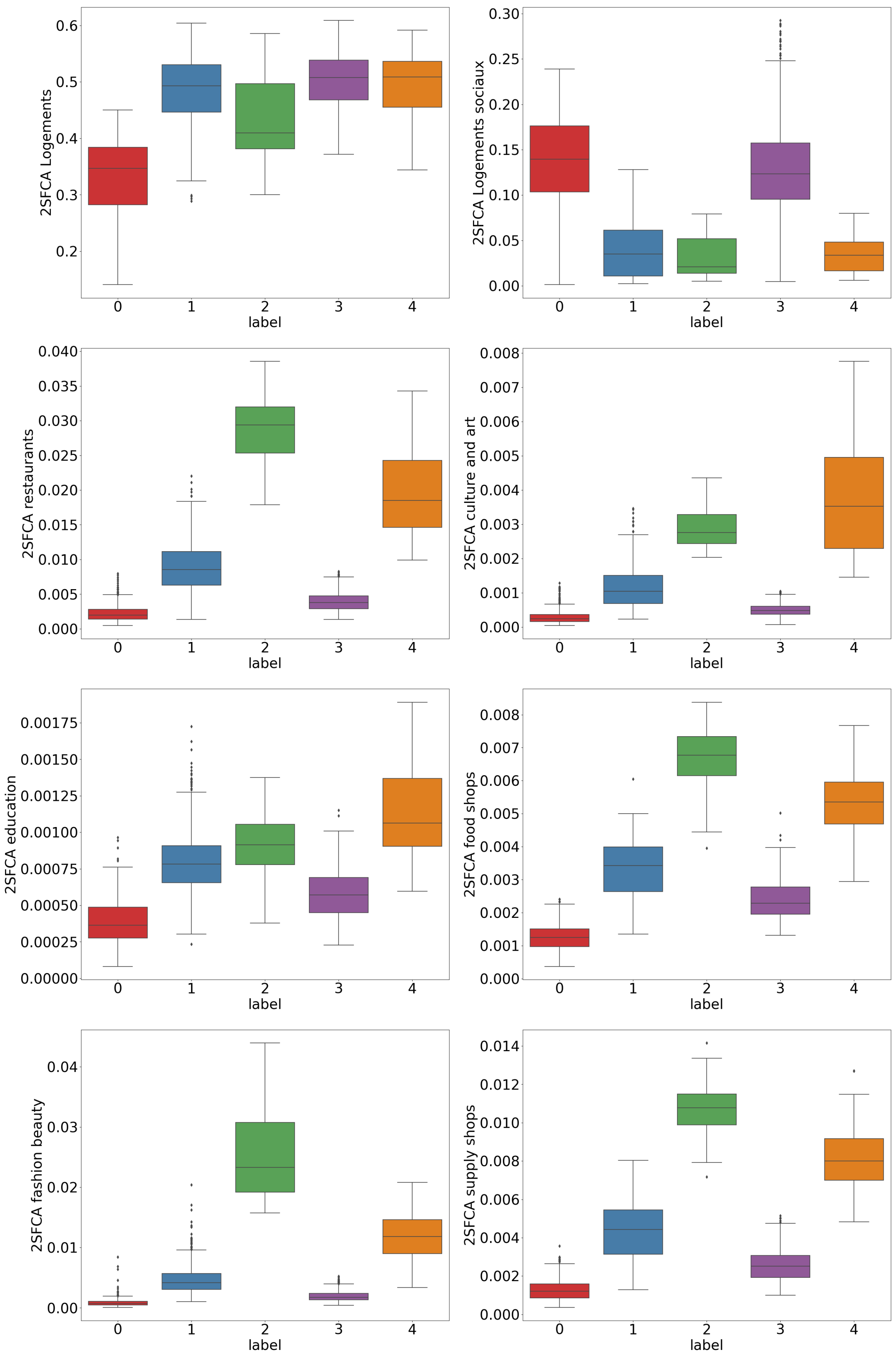

Figure S2. Box plots of the MiniBatchKMeans clusters for the different accessibility scores for Paris.

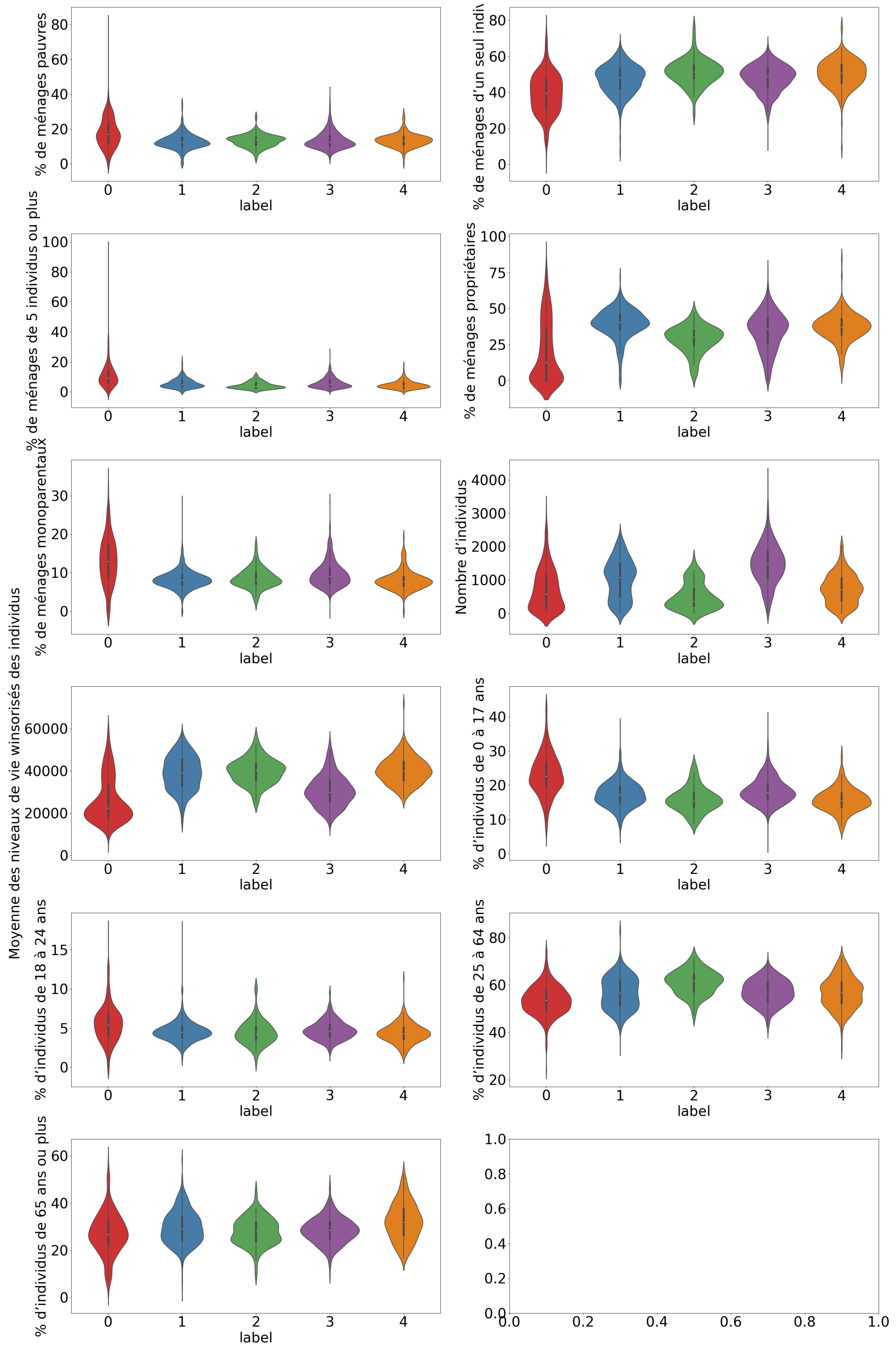

Figure S3. Violin plots of the MiniBatchKMeans clusters for different socio-economic variables for Paris.

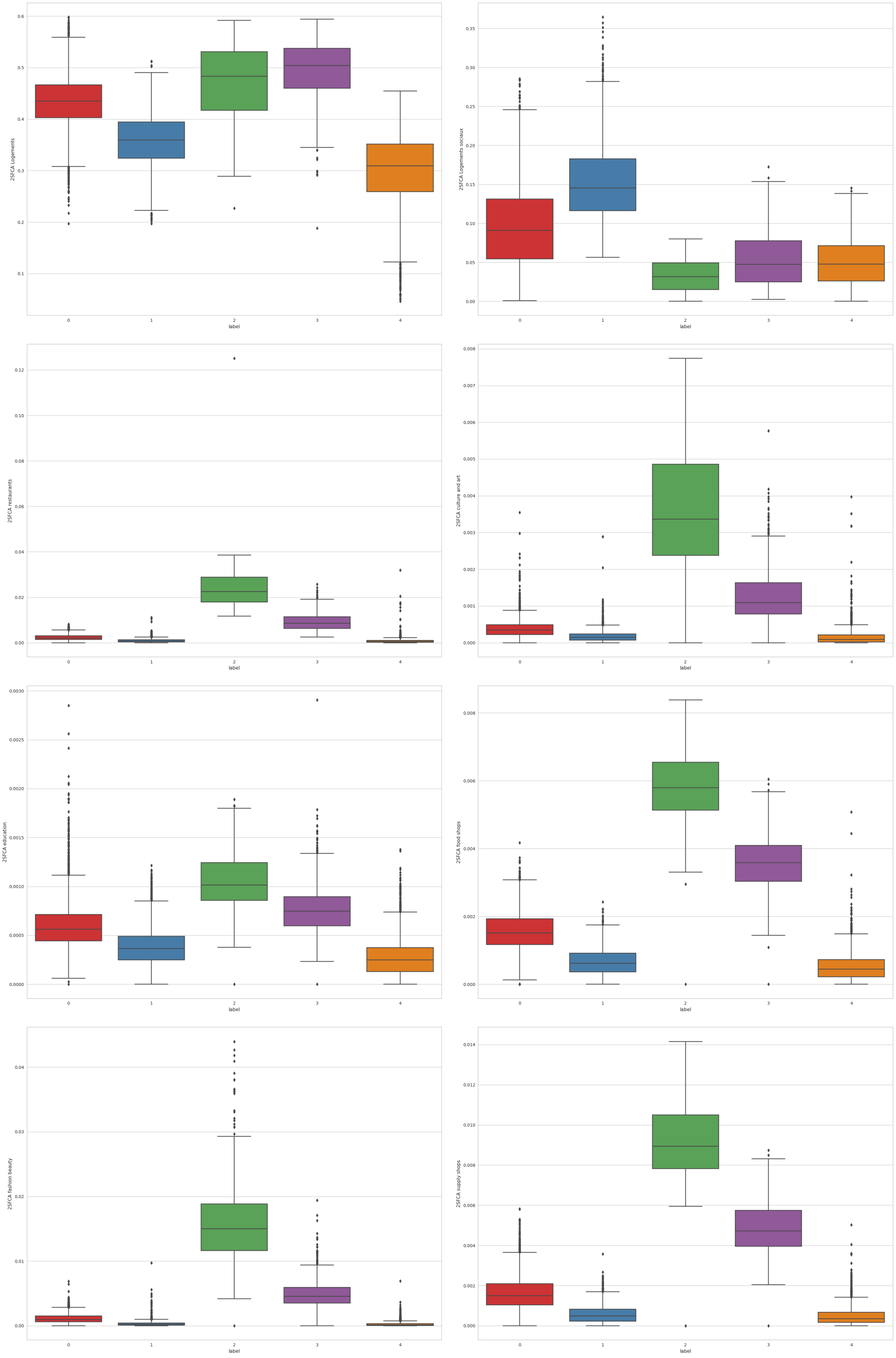

Figure S4. Box plots of the MiniBatchKMeans clusters for the different accessibility scores for the *Petite Couronne* including Paris.

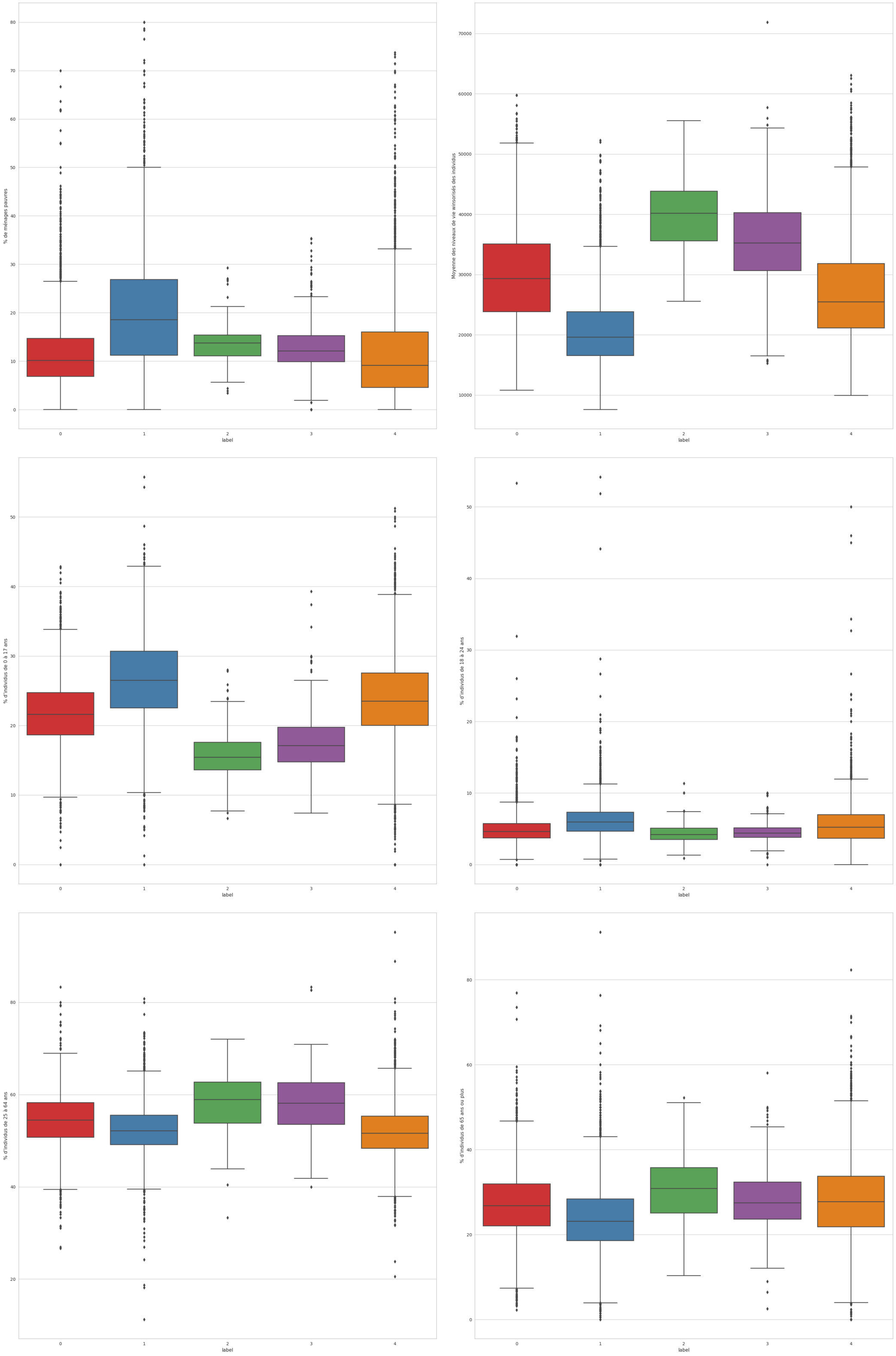

Figure S5. Violin plots of the MiniBatchKMeans clusters for different socio-economic variables for the *Petite Couronne* including Paris.

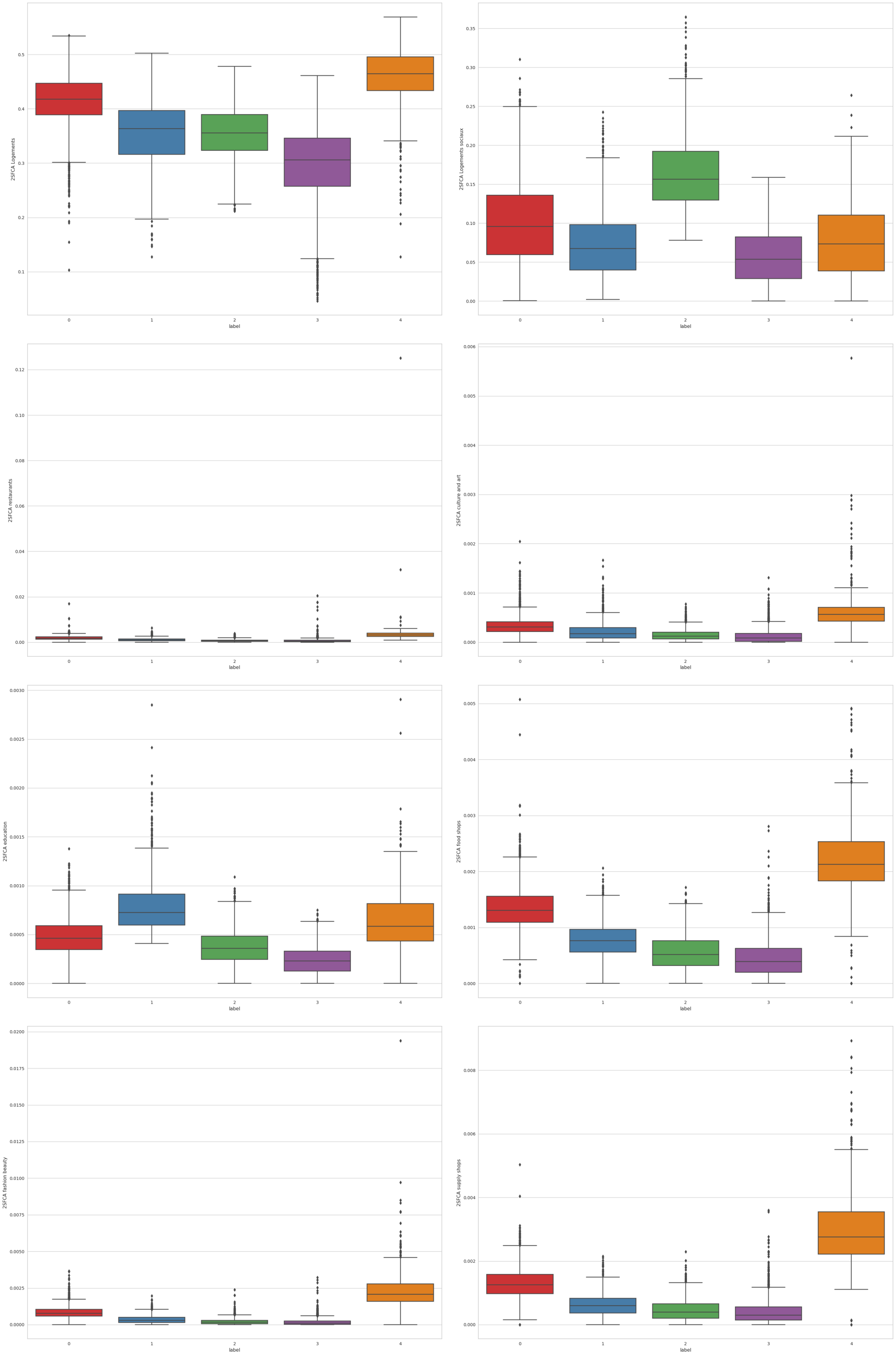

Figure S6. Box plots of the MiniBatchKMeans clusters for the different accessibility scores for the *Petite Couronne* excluding Paris.

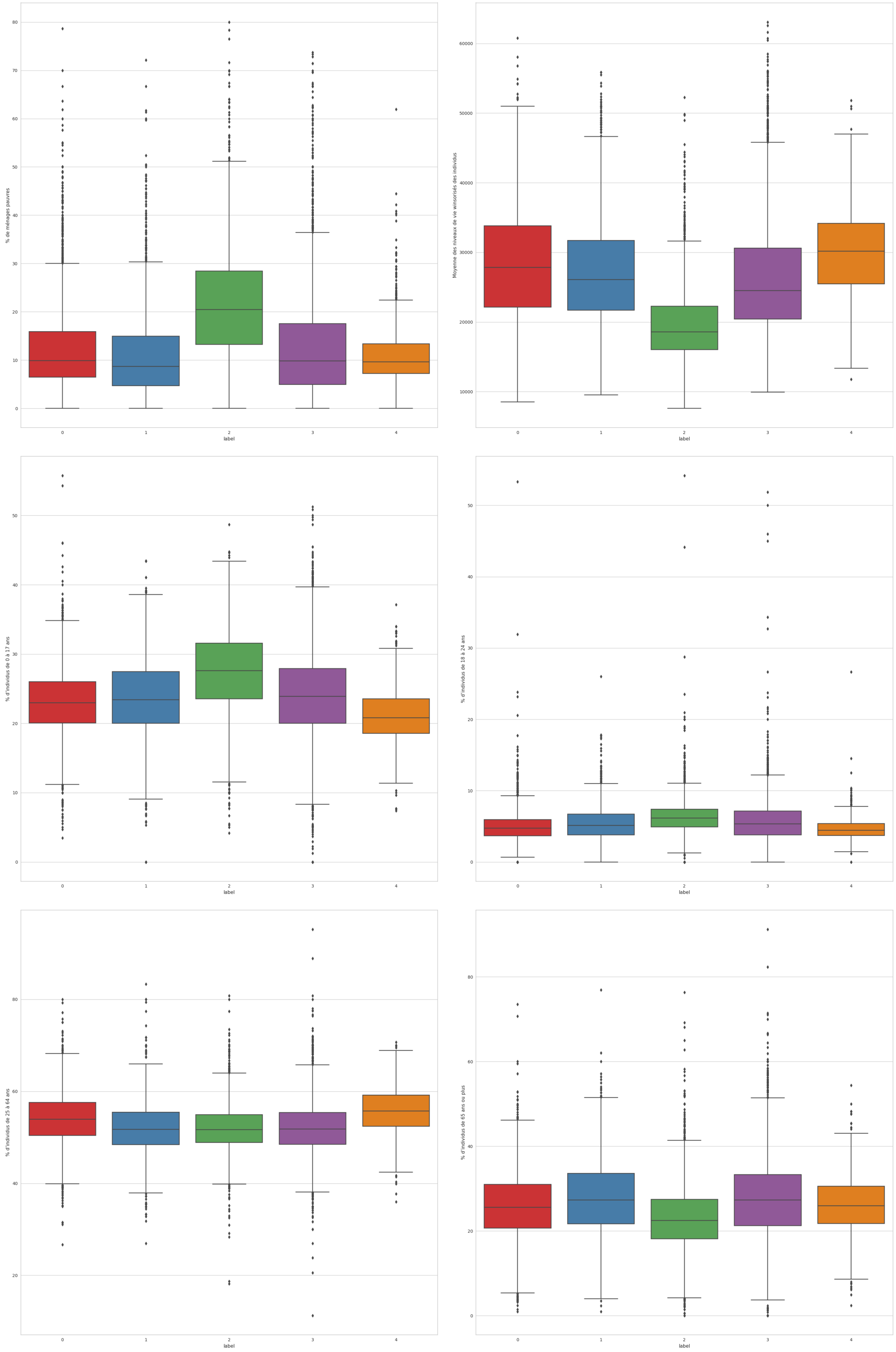

Figure S7. Violin plots of the MiniBatchKMeans clusters for different socio-economic variables for the *Petite Couronne* excluding Paris.
